# A Review of ApoE4 Interference Targeting Mitophagy Molecular Pathways for Alzheimer's Disease

**DOI:** 10.3389/fnagi.2022.881239

**Published:** 2022-05-20

**Authors:** Huiyi Chen, Feng Chen, Ying Jiang, Lu Zhang, Guizhen Hu, Furong Sun, Miaoping Zhang, Yao Ji, Yanting Chen, Gang Che, Xu Zhou, Yu Zhang

**Affiliations:** ^1^Department of Children Rehabilitation, Yuebei People's Hospital, Affiliated Hospital of Shantou University Medical College, Shaoguan, China; ^2^Guangdong Key Laboratory of Age-Related Cardiac and Cerebral Diseases, Department of Neurology, Affiliated Hospital of Guangdong Medical University, Zhanjiang, China; ^3^Department of Surgical Oncology, The First Affiliated Hospital, Zhejian University School of Medicine, Hangzhou, China

**Keywords:** Alzheimer's disease, mitophagy, mitochondrial dynamics, apolipoprotein E, neurodegenerative disease

## Abstract

Alzheimer's disease (AD) is one of the major worldwide causes of dementia that is characterized by irreversible decline in learning, memory loss, and behavioral impairments. Mitophagy is selective autophagy through the clearance of aberrant mitochondria, specifically for degradation to maintain energy generation and neuronal and synaptic function in the brain. Accumulating evidence shows that defective mitophagy is believed to be as one of the early and prominent features in AD pathogenesis and has drawn attention in the recent few years. *APOE* ε4 allele is the greatest genetic determinant for AD and is widely reported to mediate detrimental effects on mitochondria function and mitophagic process. Given the continuity of the physiological process, this review takes the mitochondrial dynamic and mitophagic core events into consideration, which highlights the current knowledge about the molecular alterations from an *APOE*-genotype perspective, synthesizes ApoE4-associated regulations, and the cross-talk between these signaling, along with the focuses on general autophagic process and several pivotal processes of mitophagy, including mitochondrial dynamic (DRP1, MFN-1), mitophagic induction (PINK1, Parkin). These may shed new light on the link between ApoE4 and AD and provide novel insights for promising mitophagy-targeted therapeutic strategies for AD.

## Introduction

Alzheimer's disease (AD) is the most common and well-known form of dementia and features age-related and progressive recognition impairment, memory loss, and learning failure, accompanied by encephalatrophy, and extracellular amyloid-β (Aβ) plaques, intracellular tangles of hyperphosphorylated Tau (Mahaman et al., 2022). Since the global aged tendency of the population has a dramatic impact on an individual and family development, and social healthcare costs throughout the world, AD with widespread prevalence has become an increasingly challenging task for elderly care, public health, and all the human beings (2020 Alzheimer's disease facts and figures, 2020). Therefore, AD targets or therapeutical strategies are urgent to be proposed currently.

It is extensively recognized that the apolipoprotein E4 (*APOE* ε4) allele remains the strongest genetic risk for sporadic AD (Belloy et al., 2020; Serrano-Pozo et al., 2021). *APOE* ε4 allele, usually existing in approximately 15% of people, lowers the age of AD onset and increases the risk of AD in a dose-dependent manner (Levy et al., 1990; Wallon et al., 2012; Ward et al., 2012; Tang et al., 2016). Accumulating difficulties remain when Aβ-targeting interference fails to effectively halt or slow clinical AD progression (Bohrmann et al., 2012; Salloway et al., 2014; Bomasang-Layno and Bronsther, 2021; Pleen and Townley, 2022); thus, it is widely believed that amyloid cascading seems to appear as less possibility for a major explanation of AD pathogenesis. Other rising insights draw more and more interests broadly. The existing investigation of *APOE* ε4 pathogenesis in AD (Norwitz et al., 2021; Serrano-Pozo et al., 2021) has expanded beyond Aβ peptide-centric mechanisms to Tau neurofibrillary degeneration, neuroinflammation (Kaur et al., 2019; Leng and Edison, 2021), synaptic loss (Zhao et al., 2020), depression (Rhodes et al., 2021; Zhang et al., 2021), blood–brain barrier disruption (Rhea and Banks, 2021; Zhang and Xie, 2021), insulin resistance (Jabeen et al., 2021), gut dysfunction (Hoffman et al., 2019), oxidative stress (Butterfield and Mattson, 2020), and autophagic deficit (Chen et al., 2021; Sohn et al., 2021; Eran and Ronit, 2022); particularly, damaged mitophagy (Simonovitch et al., 2019; Schmukler et al., 2020; Liang et al., 2021a; Morton et al., 2021; Wang et al., 2021b). However, the precise molecular mechanisms underlying ApoE4 pathogenic effects during AD have not yet been elucidated.

Mitochondria consisting of the outer mitochondrial membrane (OMM), inner mitochondrial membrane (IMM), intermembrane space, and mitochondrial matrix performs diverse essential roles in multiple intracellular homeostasis events, including tricarboxylic acid cycle, β-oxidation of fatty acids, genetic information storage, Ca^2+^ homeostasis, and biosynthesis of intermediates for cell growth or death (Moreira et al., 2010; Kauppila et al., 2017). The integrity of the mitochondrial structure is indispensable for mitochondrial function and mitophagy, which demonstrates major involvement in Aβ and Tau pathology (Parker et al., 1994; Zhu et al., 2013; Monzio Compagnoni et al., 2020). Diverse mitophagy-dependent signaling pathways have been established, many of which are conserved from *Caenorhabditis elegans* to humans (Egan et al., 2011; Fivenson et al., 2017; Kerr et al., 2017; Gustafsson and Dorn, 2019; Cai and Jeong, 2020; Wang et al., 2021b). Mitophagy, a stress-response mechanism to suppress mitochondrion-dependent apoptosis for neuronal protection (Pan et al., 2021), is fairly fundamental for mitochondrial quality control, function recovery, and self-renewal, whose deficiency is revealed in AD iPSC-derived neurons, as well as hippocampal samples from the AD model mice and patients with AD, thus receiving growing attention as a promising mechanism for AD-targeted therapy of late years (Fang et al., 2014, 2019; Xu et al., 2017; Tran and Reddy, 2020; Sukhorukov et al., 2021; Wang et al., 2021b). Specifically, accumulating evidence implies that mitophagy deficit fails to maintain mitochondrial clearance (Kerr et al., 2017), axonal transport (Ashrafi and Schwarz, 2015), and synapse biosynthesis (Pan et al., 2021), which exacerbates neuroinflammation, Aβ and p-Tau deposition, and energy dysfunction, thereby promoting AD pathology and memory loss (Song et al., 2021). Mitophagy-dependent signaling is reported to not only reduce Aβ and tau pathology (Pan et al., 2021), but also alleviate cognitive damages (Fang et al., 2019; Pan et al., 2021) through AMPK and SIRT1 activation, mTOR inhibition, and lysosomal functional improvement in AD progression (Fang et al., 2019). These evidence suggest that normal mitophagy protection or maintenance is an essential target for AD therapeutic development, while there is yet no effective and clear therapeutic approach by which to artificially manipulate mitophagy so as to improve the clinical symptoms and prolong the survival of patients affected by AD. Given its significance in various physiological processes, mitophagy has been barged to the forefront of AD researches after being studied for years. ApoE isoforms have been reported to have multiple effects of mitochondrial dysfunction, and mitophagy and ApoE4 seem to have negative impacts on diverse physiological processes (Chang et al., 2005; Chen et al., 2011; Orr et al., 2019; Horner et al., 2020; Schmukler et al., 2020; Yin et al., 2020; Liang et al., 2021b; Qi et al., 2021; Eran and Ronit, 2022). Many issues remain ambiguous and seem appealling for further investigations, however, both the functional and mechanistic multiformity of ApoE4 effects of mitophagy have emerged and been extensively enlightened in AD pathogenesis (Chen et al., 2011; Palikaras et al., 2017; Simonovitch et al., 2019; Schmukler et al., 2020; Sohn et al., 2021; Eran and Ronit, 2022).

**Figure 1:**
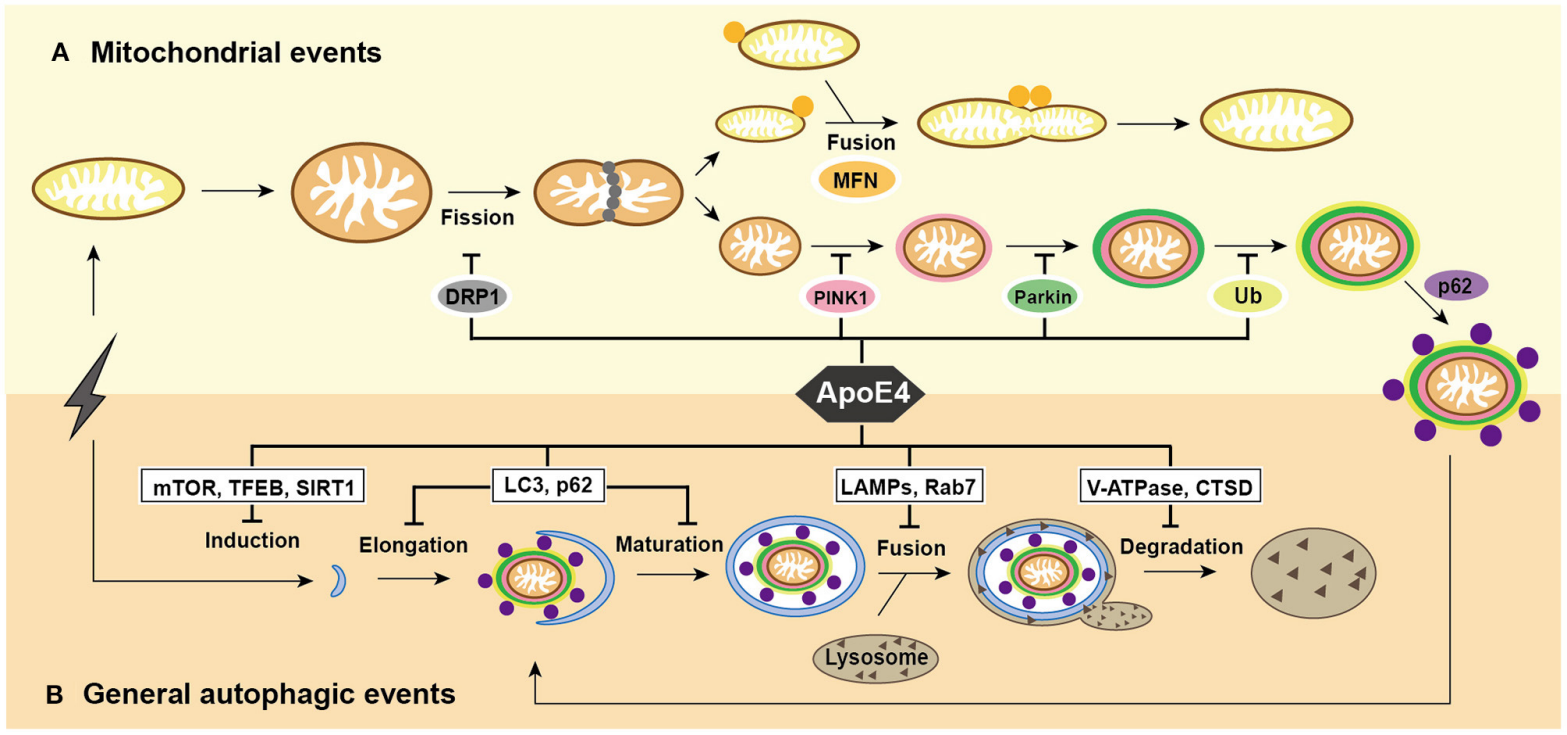
Summary of mitochondrial events and general autophagic events under ApoE4 intervention. **(A)** After excessive cellular stress, mitochondria begin to swell and fragment into healthy mitochondria for fusion, and pre-degraded mitochondria for mitophagy. damaged mitochondria induce PINK1/Parkin pathways and then participate in general autophagic flux. ApoE4 is directly or indirectly involved in mitochondrial events, including DRP1 pathways during fission; the MFNs pathways during fusion; and the PINK1/Parkin pathways during mitophagic induction. **(B)** After excessive cellular stress, the omegasome is initiated to form an elongated into a phagophore, which encloses the damaged mitochondria and becomes a mature autophagosome. Lysosomes recruit to autophagosome and fuse with it to form autolysosome, the ultimate area of mitochondrial degradation. ApoE4 intervenes autophagic initiation by the mTOR, TFEB, and SIRT1 pathways; compromises elongation by LC3, p62, and Rab5 pathways; blocks autophagosome-lysosome fusion by the Rab7, LAMP2, and LAMP1 pathways; damages lysosomal degradation by CTSD and V-ATPase pathway.

Here, we have reviewed emerging findings for the central and multi-faceted roles that mitophagy plays in the pathogenesis of AD, described physiological changes from the pre-mitophagy events to mitophagy proceeding considering the continuity of the physiological processes, integrated the signaling pathways suggesting the relationships between ApoE4 and mitophagy deficit, and discussed the underlying mechanisms that how ApoE4 triggers these abnormalities and ultimately contributes to AD pathogenesis, while highlighting potential therapeutic targets that may correct these dysfunctions (Figure 1).

## ApoE4 and General Autophagic Processes in AD

Autophagy is a ubiquitous event highly conserved from yeast to mammals and functions as a recycling system for maintaining metabolic homeostasis and cellular self-renewal (Parzych and Klionsky, 2014). Strictly, there are four main forms—macroautophagy, microautophagy, chaperone-mediated autophagy, and selective autophagy. The term “autophagy” usually means “macroautophagy”, a non-selective process. In autophagy, cargoes are labeled and enclosed by a double-membrane vesicle and become autophagosomes, then autophagosomes recruit and fuse with the lysosome to address degradation. Namely, this is highly dependent on functionally vesicular trafficking (Parzych and Klionsky, 2014). Relatively, selective autophagy is when several specific organelles, such as mitochondria, lipids, and endoplasmic reticulum (ER), fulfill their degradation by the core machinery of autophagy (Scrivo et al., 2018). Mitophagy is a selective mode of autophagy targeted engulfment and destruction of mitochondria, namely, mitophagy shares several common mechanisms with autophagy.

Autophagy mediates the degradation of abnormal cellular components or damaged organelles, closely associated with ontogeny and growth, oxidative damage protection, the malignant proliferation of tumor cells, and neurodegenerative diseases (Parzych and Klionsky, 2014). It is extensively believed autophagic impairment in AD and *APOE* genotypes plays diverse roles in the underlying mechanisms (Chen et al., 2021). Specifically, ApoE4 mainly inhibits autophagic initiation by the upregulation of the mammalian target of rapamycin (mTOR), the binding to transcription factor EB (TFEB), the inhibited expression of Surtuin 1 (SIRT1), and forkhead box O3a (FOXO3a), and their signaling pathways, respectively (Theendakara et al., 2016; Parcon et al., 2018; Lima et al., 2020; Sohn et al., 2021); thereby interfering with phagophore elongation and completion, which is involving PI3K, LC3, p62, and Rab5 (Gilat-Frenkel et al., 2014; Ong et al., 2014; Simonovitch et al., 2016; Nuriel et al., 2017; Parcon et al., 2018; Li et al., 2019), blocking autophagosome-lysosome fusion by lysosomal associated membrane protein 1 (LAMP1), LAMP2, and Rab7-dependent movement (Nuriel et al., 2017; Parcon et al., 2018) and damages phospholipid homeostasis and lysosome membrane integrity (Ji et al., 2002; Zhu et al., 2015; Persson et al., 2017). This compromises lysosomal degradation by the leakage of cathepsin D (Ji et al., 2002; Belinson et al., 2008; Nuriel et al., 2017; Persson et al., 2017) and the abnormal lysosomal acidification mediated by the proton pump V-ATPase (Nuriel et al., 2017; Larramona-Arcas et al., 2020). As a part of these general processes of autophagy—initiation, elongation, autophagosome-lysosome fusion, and lysosomal degradation—overlapped with those of mitophagy and they share common mechanisms, namely, it is probably that ApoE4 also impedes mitophagy by these common processes.

We have summarized ApoE4 regulation on the stages shared by mitophagy and autophagy and illustrated the insights underlying AD-targeted therapy, then we will discuss the association between ApoE4 and mitophagy from the perspective of mitophagy-exclusive processes, including mitochondrial dynamic equilibrium (fission and fusion), and mitophagy-specific induction.

## ApoE4 and Mitophagy-Specific Processes in AD

Mitochondria is a highly dynamic organelle that can form long tubular networks for highly interconnected networks, and continually undergoes the cycle of fusion and fission in response to environmental changes (Wolf et al., 2020). When mitochondria are damaged, the swelling mitochondria trigger component redistribution and are divided into relatively healthy mitochondria for fusion and damaged mitochondria for mitophagy and degradation. After fission events, depolarized mitochondria induce PTEN-induced putative kinase 1 (PINK1)/Parkin-dependent mitophagy (Wolf et al., 2020; Luan et al., 2021). As mitochondrial events are closely linked and occur in succession, mitochondrial fission and fusion are disrupted followed by aberrant mitophagy.

### ApoE4 and Mitochondrial Fission in AD

Mitochondrial fission is required for mitophagy and mitochondrial transport, while fusion prevents mitochondria from undergoing mitophagy by replenishing mitochondrial DNA and promoting biolipid exchange (Wolf et al., 2020). Mitochondria fission is a basic and vital process *via* programmed and sequential membrane movement that (Mahaman et al., 2022) involves healthy mitochondria fragments for growing physiological requirements; (2020) aged or damaged mitochondria divided into healthy mitochondria for recycling and pre-degraded mitochondria for clearance (Wolf et al., 2020; Luan et al., 2021). Impaired mitochondrial dynamics is widely established in AD model mice and cells, as well as AD individuals (Manczak et al., 2011; Reddy et al., 2011, 2018; Manczak and Reddy, 2012a; Zhu et al., 2013; Kandimalla et al., 2021), as determined by the lower expression of mitochondrial fission genes (*DRP1* and *FIS1*) and higher phosphorylation level of dynamin-related protein1 (DRP1), leading to reductive mitochondria fragmentation and neuronal energy dysfunction (Reddy et al., 2011; Manczak and Reddy, 2012a; Misrani et al., 2021; Wang et al., 2021a; Dhapola et al., 2022). Incidentally, additional studies indicate that decreased DRP1 promotes cognitive performance and improves mitophagy and dendritic spines in tau-transgenic mouse model and APP/PS1 mice (Kandimalla et al., 2021; Misrani et al., 2021; Song et al., 2021), in which DRP1 is overactivated under abnormal conditions; and consistently, various DRP1 inhibitors promote fusion and improve cognition in AD (Dhapola et al., 2022). These suggest that correcting DRP1 activity might confer tolerance to cytotoxicity from p-Tau and Aβ (Manczak and Reddy, 2012a; Kuruva et al., 2017; Reddy and Oliver, 2019) and there is a need for further investigation into the identified regulation underlying AD events.

**Figure 2:**
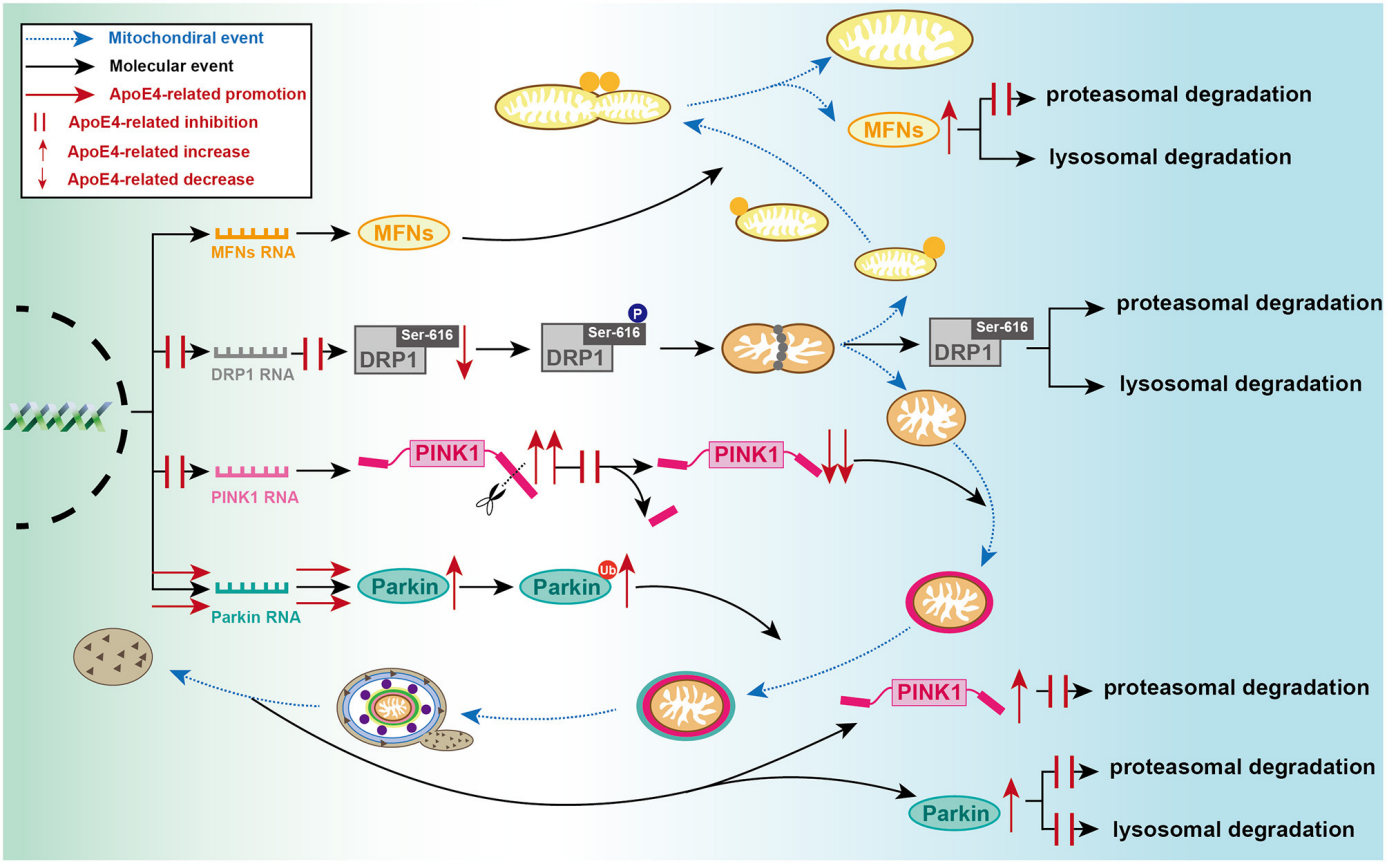
Detailed processes of established ApoE4-mediated regulation of mitochondrial events at molecular level. During mitochondrial fusion, ApoE4 interferes with MFNs proteasomal degradation, then leading to MFN dynamic dysfunction. During mitochondrial fission, ApoE4 inhibits DRP1 transcription and expression, thus leading to a lower capacity of fission. During mitophagic induction, AcpoE4 inhibits PINK1 transcription and impairs proteolytic cleavage of FL-PINK1, exemplified by FL-PINK1 elevation and cleaved-PINK1 reduction. ApoE4 also blocks PINK1 proteasomal degradation, resulting in PINK1 dynamic dysfunction. ApoE4 not only promotes Parkin transcription and expression but also disrupts both proteasomal and lysosomal degradation, leading to a remarkable increase of ubiquitinated Parkin and total Parkin.

DRP1 GTPase and its receptors (FIS1, MFF, MID49, and MiD51/MIEF1) are the most broadly investigated fission regulators network in recent years (Chan, 2012). Mitochondrial fission is promoted by DRP1-Ser616 phosphorylation and inhibited by DRP1-Ser637 phosphorylation (Taguchi et al., 2007; Cereghetti et al., 2008; Chang and Blackstone, 2010). DRP1 translocation induces mitochondrial dynamics disturbance and contributes to the imbalance of mitochondrial dynamics toward fission, consequently triggering mitophagy and inhibiting pathogen-induced apoptosis (Schmukler et al., 2020). ApoE3 is the most common isoform found among people, thus usually functioning as the control group during ApoE4 studies (Liu et al., 2013). Compared to ApoE3, ApoE4 inhibits *DNM1L* transcription and attenuates DRP1 expression in astrocyte cell lines (Schmukler et al., 2020), primary astrocytes, and hippocampus from *ApoE*-TR mice (Yin et al., 2020), and the ostmortem brain specimens from *APOE* ε4 carriers and patients with AD carrying *APOE* ε4/4 (Simonovitch et al., 2019; Yin et al., 2020). DRP1 level is not different in ApoE3 and ApoE4 astrocytes with the treatment of lysosomal inhibitor chloroquine or proteasomal inhibitor MG-132, indicating that DRP1 lysosomal and proteasomal degradation pathways are not affected by *APOE* genotype (Schmukler et al., 2020). ApoE4 attenuates carbonyl cyanide m-chlorophenyl hydrazone (CCCP)-induced increase of DRP1 in ApoE4-astrocytes compared to ApoE3, indicating an impaired stress reaction to mitochondrial energy disorders (Schmukler et al., 2020). These results suggest that decreased DRP1 level may majorly result in compromised RNA synthesis induced by ApoE4 (Figure 2), instead of enhancing lysosomal and proteasomal degradation (Gomes et al., 2011; Rambold et al., 2011; Simonovitch et al., 2019; Schmukler et al., 2020). However, other studies display inconsistent observations. ApoE4 (Δ272–299), an effective fragment derived from ApoE4, promotes MFF expression and DRP1-Ser616 phosphorylation as well, and compromises mitofusin (MFN)−1 and−2 and optic atrophy 1 (OPA1) expression *in vitro*, indicating that ApoE4 (Δ272–299) possess pro-fission activation and fusion inhibition (Liang et al., 2021b). These contradictory observations probably are by virtue of the effects of different structural features. It is well-known that ApoE4 detrimental pathogenic effects are highly dependent on its peculiar conformation with a more compacted structure and higher oligomerized inclination, which is resulted from two amino acid residues varying from ApoE3 (Chen et al., 2011). Accordingly, ApoE4 is more susceptible to cleave than ApoE3 (Huang et al., 2001; Kothari et al., 2021). Different ApoE4 fragments differentially couple with certain neurotoxicity and induce mitochondrial disorders (Chang et al., 2005). Both the lipid-binding region (amino acids 241–272) and receptor-binding region (amino acids 135–150) are indispensable for ApoE4 neurotoxicity, and the amino acids 273-299 are identified for its neuroprotection (Chang et al., 2005). Only ApoE4 fragments with these two regions can escape from the secretory pathway and interact with mitochondria, thus impairing mitochondrial function and integrity (Chang et al., 2005). Briefly, in our views, concerning the comprehensive effects of integrated protein structure, full-length ApoE4, instead of its (Δ272–299) fragment, is proposed to play primary roles to inhibit fission by DRP1 expression, of course, there is still appealed for further investigation in the complicated potency and the prime functional regions. Incidentally, glucose-regulated protein 75 (GRP75) inhibitor is effective to correct the pro-fission activation induced by the ApoE4 (Δ272–299) (Liang et al., 2021b), indicating a promising insight for AD-targeted therapy.

Owing to a lack of detailed investigation into DRP1 properties, it is also worth determining the DRP1 activity changes from the ApoE4 perspective or evaluating mitochondrial fission from other perspectives. Although the regulatory effects of ApoE4 on fission have been established, the alternative mechanisms that how ApoE4 affects DRP1 function in addition to transcription remain unknown, and the potential signaling interactions are here summarized. First, ApoE4 exerts multiple inhibitions on SIRT1 dynamics and function (Theendakara et al., 2013, 2016), then resulting in disturbance of SIRT1-mediated mitochondrial fragmentation in mitophagy (Qiao et al., 2018). Specifically, in both the *in vivo* and *in vitro*, ApoE4 interferes with *SIRT1* transcription, expression, and enzyme activity more than ApoE3 (Lattanzio et al., 2014; Theendakara et al., 2016) and the inhibited effects seem to result from a pretranscriptional affect—the specific binding of ApoE4-*SIRT1* promoter with the highest affinity among ApoE isoforms (Theendakara et al., 2016; Lima et al., 2020). Moreover, only ApoE4 triggers SIRT1 mislocalization to the cytoplasm and impairs the SIRT1 function (Theendakara et al., 2016; Lima et al., 2020). Notably, overexpressed SIRT1 can rescue ApoE4-induced physiopathologic alterations in AD events (Theendakara et al., 2013). Since SIRT1-involved neuroprotection plays extensive and multiple roles in AD progression (Fujita and Yamashita, 2018; Gomes et al., 2018), it might be helpful to extract the relationship between SIRT1 and ApoE4 and the potential mechanisms. Second, the abnormal interactions between DRP1 and hyperphosphorylated Tau, as well as DRP1 and Aβ monomers and oligomers, are established by coimmunoprecipitation (co-IP) and double-labeling immunofluorescence analysis, in postmortem brain tissues of AD patients and brain homogenates from several AD mouse models (Manczak et al., 2011; Reddy et al., 2011; Manczak and Reddy, 2012a; Abtahi et al., 2020; Kandimalla et al., 2021), which is speculated that excessive p-Tau and Aβ accumulation may synergistically disrupt mitochondrial fragmentation in a ApoE4-dose-dependent manner. These findings may help to shed new light on the alternative link between ApoE4 and mitophagic dynamics.

Taken together, these findings suggest that ApoE4 disrupts mitochondrial fission by the inhibition of DRP1 transcription and expression (Schmukler et al., 2020), DRP-Aβ interaction (Manczak et al., 2016), DRP1-Tau interaction (Kandimalla et al., 2016), and the downregulation of its regulators SIRT1 level (Theendakara et al., 2013), ultimately resulting in dysregulated mitochondrial dynamics, consequently probably triggering irreversible and progressive neuronal damage and cognitive impairment (Manczak and Reddy, 2012a). These seem to reveal a new hypothesis for neurodegeneration in AD involving mitochondrial dynamics and ApoE4-targeted pathology at the molecular level, leading to a better understanding of the mechanisms underlying the ApoE4 regulatory effect on mitophagy in AD.

### ApoE4 and Mitochondrial Fusion in AD

Mitochondrial fusion includes two types IMM fusion is facilitated by OPA1 and OMM fusion is majorly dependent on the oligomerization of MFN-1 and−2 (Eisner et al., 2018; Tilokani et al., 2018; Dhapola et al., 2022). MFN-1 and−2 are transmembrane GTPases embedded in OMM that are usually degraded by proteasomes to prevent mitochondrial fusion and maintain mitochondrial fission (Nguyen et al., 2016; Meng et al., 2019). Mitochondrial fusion is established associations with AD pathogenesis as determined by the mitochondrial dynamic proteins that influence AD cognitive function in a dose-dependent manner (Yin et al., 2020). Interestingly, some reports indicate increased levels of MFN-1, MFN-2 and OPA1 are detected in hippocampal tissue of patients with AD and APP/PS1 mice (Wang et al., 2009, 2021b; Song et al., 2021), while another study found decreased levels of mitochondrial fusion genes (*MFN-1, MFN-2, TOMM40*, and *OPA1*) in the frontal cortex tissue from AD patients (Reddy et al., 2011), which may be explained by the samples from different brain region and appeals for further investigation and reconfirmation in detail Although the definite alterations and impacts remain contradictory and ambiguous, at least, there are some associations between mitochondrial fusion and AD pathogenesis to a certain extent.

The mitochondrial network is hyperfused in ApoE4 astrocytes (Schmukler et al., 2020). ApoE4 elevates MFN-1 protein level in N_2_a cells (Orr et al., 2019), astrocytes cell lines (Schmukler et al., 2020), primary astrocytes, and brain tissues from young 5-month-old *APOE* ε4-TR mice (Simonovitch et al., 2019). However, the expression of MFN-1 and MFN-2 have significantly decreased in the postmortem brain tissues of *APOE* ε4 carriers with a mean age of 84.6 years (Yin et al., 2020). The contradictory results may be involved in a discrepancy of different ages or objects. For instance, the MFN-1 level is decreased in AD patients with a mean age of 88.5 years (Yin et al., 2020), while the levels of MFN-1, MFN-2, and OPA1 are increased in the hippocampus of patients with AD with the age range from 60 to 89 years, in which the mean age is lower (Wang et al., 2009). Specifically, in young subjects, MFN production normally remains and is able to function compensatory in response to ApoE4-induced damage, thus majorly demonstrating degradation blockage exemplified by MFN elevation (Orr et al., 2019; Simonovitch et al., 2019; Schmukler et al., 2020). With increasing age, age possesses more profound and irreversible degenerated potency on overall cellular metabolisms (Mkrtchyan et al., 2020; Wissler Gerdes et al., 2020) and the compensatory capacity may have been exhausted. Thus, old subjects majorly demonstrate deficient production exemplified by MFN reduction. It may be helpful to study MFN-1 changes under the specific conditions, such as in *APOE* ε4/4/AD patients or animal models. Despite the inconsistent results, there are certain association between ApoE4 and mitochondrial fusion proteins. Additionally, compared to ApoE3 astrocytes, the increase of total MFN-1 and ubiquitinated MFN-1 are lower in APOE4 astrocytes without transcriptional change, while it is found the consistent alteration under MG-132 treatment (Schmukler et al., 2020). These results indicate ApoE4 induces MFN-1 accumulation and defective degradation without proteasomal inhibition (Figure 2), and decreases MG-132-triggered accumulation and degraded response (Schmukler et al., 2020). Namely, MG-132 blocks MFN-1 elimination in ApoE3 astrocytes but functions less in the ApoE4 setting. ApoE4 has probably compromised the degraded machinery before MG-132 treatment or eliminated the potency of inhibitor, which appears that ApoE4 effects may be equivalent to those of MG-132, the inhibition of MFN-1 degradation and ubiquitination in young mice. Moreover, CCCP-induced reduction of MFN-1 is lower in ApoE4 astrocytes than those of ApoE3 (Schmukler et al., 2020), indicating an impaired stress reaction as well. Therefore, ApoE4 may play significant roles in degraded machinery and mitochondrial fusion, or in certain steps before fusion so that MG-132 and CCCP fail to trigger corresponding stress signaling in the ApoE4 setting.

Like DRP1, the interfered effects of ApoE4 on MFN-mediated fusion are approximately established, but the regulatory mechanism in which APOE4 affects MFN metabolism is yet unclear, and several potential mediators are illustrated as follows. First, the levels of fusion proteins (MFN-1, MFN-2, OPA1) are growing and the polyubiquitinated level of MFN-2 is reduced, while the fission proteins (FIS1 and GLP1) are not distinctively altered in HEK293 cells overexpressing human Tau, and rat primary hippocampal neurons (Li et al., 2016), and the brain tissues from human Tau-transgenic mice (Kandimalla et al., 2016), indicating MFNs accumulation and mitochondrial dysfunction caused by human Tau. Second, excess Ca^2+^ in cytoplasm triggered by ApoE4 (Larramona-Arcas et al., 2020) partially blocks GTP-mediated oligomerization of MFN-1, thus resulting in impaired complex formation for fusion (Ishihara et al., 2017). Third, functional factors in the mitochondrial network link with each other by various chemical modifications. MFN-2 is phosphorylated by PINK1 for Parkin recruitment (Holness and Sugden, 2003; Bhupana et al., 2020; Du et al., 2021) and is ubiquitinated by Parkin (Zachari and Ktistakis, 2020), while PINK1 and Parkin are collectively regulated by ApoE4 (Simonovitch et al., 2019; Schmukler et al., 2020; Sohn et al., 2021).

Briefly, ApoE4 seems to interfere with mitochondrial fusion by blocking proteasomal degradation of MFN, and other potential regulations are required for further confirmation. Considering fission alteration, ApoE4-mediated fission failures probably couple with ApoE4-mediated fusion abnormalities and jointly deteriorate mitochondrial dynamics imbalance. It may be interesting to investigate whether there is a mutual implication between MFN-1 upregulation and DRP1 downregulation during ApoE4-impaired mitophagy. Overall, these results consistently suggest that the ApoE status specifically alters mitochondrial fission and fusion possibly *via* DRP1 and MFN-1 signaling, and the precise underlying mechanisms may be intriguing to be identified at different levels and by different measuring methods, such as activity, conformation, and chemical modification.

### ApoE4 and Mitophagy Induction in AD

Mitophagy usually occurs in a ubiquitin-dependent manner, which is mediated by the classical PINK1/Parkin signaling pathway (Narendra et al., 2012), or in a receptor-mediated manner, which is independent of ubiquitin and regulated by NIX/BNIP3 (Sandoval et al., 2008; Li et al., 2021), FUNDC1 (Narendra et al., 2012; Chen et al., 2016), and BCL2-L-13 (Yamaguchi et al., 2016; Guan et al., 2018). Normally, PINK1 enters the mitochondria *via* translocase of outer mitochondrial membrane 40 (TOMM40) machinery, then is cleaved by PARL in a mitochondrial membrane potential (MMP)-dependent manner followed by degradation (Jin et al., 2010; Matsuda et al., 2010; Gottschalk et al., 2014). Upon mitochondrial depolarization, MMP collapse intervenes TOM complex thus leading to PINK1 accumulation on OMM and its autophosphorylation and dimerization (Jin et al., 2010; Narendra et al., 2012; Zachari and Ktistakis, 2020). PINK1 triggers the phosphorylation of ubiquitin, Parkin, and MFN-2, and couples with phosphorylated ubiquitin to facilitate Parkin recruitment. Activated Parkin further conjugates ubiquitin and boosts the ubiquitination of MIRO, voltage-dependent anion channel 1 (VDAC1) and MFNs on mitochondria (Zachari and Ktistakis, 2020), in which the ubiquitin chains can recruit p62, OPTN, NDP52, TAX1BP1, and NBR1 and facilitate mitophagy (Schmidt et al., 2021).

#### ApoE4 and PINK1/Parkin in AD

The PINK1-Parkin signaling pathway is one of the well-described molecular mechanisms inducing mitophagy (Narendra et al., 2012). Both PINK1 and Parkin have been widely investigated not only in PD but also in AD (El Gaamouch et al., 2016; Martín-Maestro et al., 2016). The levels of PINK1 and Parkin, as well as the related factors, are usually remarkably increased *in vivo* and *in vitro* models of AD and patients with AD (Mise et al., 2017; Ochi et al., 2020; Goudarzi et al., 2021), indicating various mitochondrial disturbances or dysfunctional mitophagy. However, the additional finding indicates upregulation of PINK1-Parkin-mediated mitophagy signaling improves cognitive behavior and memory in rats injected with Aβ_1−42_ (Han et al., 2020). The elevation does not necessarily mean ongoing and activated mitophagy or a determined change in mitochondria status, as it may be caused by blocked degradation, available compensatory effect in response to various dysfunction in early AD progression, or exhausted compensatory storage along with aging in the terminal stage.

ApoE4 decreases MMP and ATP storage (Schmukler et al., 2020). Accordingly, lower cleaved PINK1 levels and higher full-length PINK1 (FL-PINK1) levels are found in ApoE4 astrocytes and hippocampal neurons from *APOE* ε4-TR mice (Simonovitch et al., 2019) (Figure 2), indicating the impaired proteolytic cleavage of PINK1 is in response to ApoE4-induced mitochondrial dysfunction (Figure 2). Incidentally, both the RNA level in *APOE* ε4 astrocytes and the total protein level of PINK1 in *APOE* ε4 carriers are decreased (Schmukler et al., 2020; Sohn et al., 2021), as well as PINK1-mediated phosphorylation of ubiquitin in *APOE* ε4 carriers is significantly suppressed (Sohn et al., 2021), suggesting inhibited expression of PINK1 and damaged potential reserve capacity of mitophagy. Taken together, it seems to reveal that under the ApoE4 condition, the proportion of FL-PINK1 is increased and the proportion of cleaved PINK1 is accordingly decreased at the basic of the reductive total amount of PINK1. These observations echo the alterations in AD abovementioned (Mise et al., 2017; Ochi et al., 2020; Goudarzi et al., 2021). Additionally, ApoE4 rather than ApoE3 astrocytes demonstrate lower level of PINK1 upregulation caused by MG-132 (Schmukler et al., 2020), similarly indicating an impaired degradation caused by ApoE4. However, an alternative study reported the level of the FL-PINK1 does not differ in the hippocampus of ApoE3 and ApoE4 mice (Simonovitch et al., 2019). Despite either FL-PINK1 is increased or unchangeable, the relation between PINK1 and ApoE4 is gradually established (Schmukler et al., 2020).

With enhancing *PARK2* mRNA synthesis (Schmukler et al., 2020), the total amount of Parkin and ubiquitinated Parkin levels are concomitantly dramatically increased in different hippocampal sections from *APOE* ε4-TR mice (Simonovitch et al., 2019) and ApoE4 astrocytes (Schmukler et al., 2020) (Figure 2). Following either the treatment with MG-132 or chloroquine, ApoE4 astrocytes display less increase of Parkin accumulation than those in ApoE3 astrocytes (Schmukler et al., 2020), indicating Parkin elevation is not only accounts for activating transcription, but its reduced proteasomal and mitophagic degradation, respectively (Figure 2). Similarly, accumulation of ubiquitinated Parkin caused by MG-132 only presents in ApoE3 astrocytes, indicating ubiquitinated Parkin also participates in proteasomal degradation and reconfirming the degradation blockage caused by ApoE4. This result is consistence with another finding regarding deficient degradation that ApoE4 directly interacts with lysosomal membranes, disrupts lysosomal acidification, and inactivates lysosomal hydrolase (Ji et al., 2002; Belinson et al., 2008; Zhu et al., 2015; Nuriel et al., 2017; Persson et al., 2017; Fote et al., 2022). It will be intriguing to examine the changes of mitophagic inducers and inhibitors on ApoE4-related pathology *in vivo*. Remarkably, autophagic inducer rapamycin has no effect on cleaved PINK1 level, but elevates FL-PINK1 level by enhancing PINK1 mRNA synthesis, decreasing MFN-1 and Parkin levels without transcriptional inhibition, and facilitates the PINK1/Parkin pathway in the ApoE4 astrocytes (Schmukler et al., 2020), leading to restoring mitochondrial metabolism, and correcting the altered medium pH without affecting cell number (Schmukler et al., 2020). These changes suggest that downstream Parkin accumulation follows upstream PINK1 deficiency, and rapamycin promotes mitophagy and remains its protective effects under ApoE4 condition, and it is likely to gain more attention as an AD therapeutical target.

Briefly, conservative speculation is that ApoE4 impedes mitophagy throughout protein synthesis and degradation, and there seems to exist a theoretical relationship between ApoE4, PINK1/Parkin-mediated mitophagy, and AD, which suggests an instructive hypothesis for neurodegenerative changes in AD involving various processes, including expression, cleavage, recruitment, ubiquitination, and degradation of PINK1 and Parkin at the molecular level. Nevertheless, the precise molecular mechanisms by which ApoE4 alters PINK1 and Parkin function have not yet been elucidated, and it may involve a complicated regulated network of mitochondrial physiological events. Herein, we present the potential machinery focusing on several pivotal mitophagic factors as follows:

#### ApoE4 and the Regulators of PINK1/Parkin in AD

FOXO3a served as a transcription factor highly represented in the brain and a new target of AD diagnosis (Pradhan et al., 2020), that maintains cellular homeostasis against stresses for neuronal protection, and regulates the transcriptional activity of autophagy (*BECN1, ATG12, PIK3C3*) and mitophagic genes (*BNIP3* and *PINK1*) (Greer and Brunet, 2005; Murtaza et al., 2017; Cheng, 2019). Akt-involved phosphorylation of FOXO3a keeps exclusion from the nucleus and inhibits its transcriptional activity (Greer and Brunet, 2005). The lower protein level of FOXO3a and the higher phosphorylation level of FOXO3a-Ser253 are found in the postmortem human brain tissues from *APOE* ε4 carriers than non-carriers (Sohn et al., 2021), indicating that the transcriptional activity and expression of FOXO3a are considerably suppressed by *APOE* ε4 allele. Consistently, this is also supported by the results from immunoblot and correlation analysis that there is a concomitantly reductive transcription of its downstream genes (*BECN1, ATG12, BNIP3*, and *PINK1*) in *APOE* ε4 carriers. Collectively, ApoE4 mediated full-scale inhibition of mitophagy *via* FOXO3a signaling, including its expression, activity-dependent on chemical modification, and the induction of its downstream effects.

SIRT1 is a NAD-dependent deacetylase that is involved in the regulation of autophagy and mitophagy (Lee et al., 2008; Salminen and Kaarniranta, 2009; Jang et al., 2012; Ou et al., 2014) and displays neuroprotective implications in AD (Julien et al., 2009; Gomes et al., 2018). SIRT1 directly binds to the transcription factor FOXO3a and mediates FOXO3a deacetylation (Motta et al., 2004; Eijkelenboom and Burgering, 2013; Ou et al., 2014). The SIRT1-FOXO3a-BNIP3 axis is indispensable for the induction of PINK1-Parkin-dependent mitophagy (Yao et al., 2021). SITR1 activation elevates Parkin expression, thus facilitating Parkin-dependent mitophagic induction (Qiao et al., 2018). Given that ApoE4 interferences of SIRT1 function have been mentioned above, it may be attributable to the lower capacity reserve of mitophagy in AD progression. The neuroprotection of overexpressed SIRT1 indicates a promising target for AD.

BNIP3, A BH3-only member of the BCL2 family operating as a mitophagic receptor on OMM (Gao et al., 2020), stabilizes PINK1 protein onto mitochondria to boost PINK1-mediated mitophagy in hypoxic conditions (Zhang et al., 2016; Zachari and Ktistakis, 2020). Performed with mass spectrometry analysis and co-IP assay, FL-PINK1 is shown interact with BNIP3, and the BNIP3-PINK1 binding disrupts proteolytic cleavage of PINK1 to maintain the stabilization of FL-PINK1 for more Parking recruitment (Zhang et al., 2016). Moreover, BNIP3-mediated mitophagy is required for FOXO3a protections against temozolomide-induced DNA double-strand breaks (He et al., 2021), while FOXO3a interacts with the *BNIP3* upstream promoter and elevates BNIP3 expression (Lu et al., 2014). These results suggest that BNIP3 function as a hinge during mitophagy. Moreover, ApoE4 attenuates the expression of BNIP3 and PINK1 *via* Foxo3 signaling in *APOE* ε4 carriers (Sohn et al., 2021), thus impairing the responsiveness of mitophagy. This may enlighten an overlooked hinge for key mitophagic induction pathways, which closely involved PINK1-induced signaling and FOXO3a-induced signaling.

Sirtuin 3 (SIRT3) is a deacetylase, highly expressed in high-metabolic tissues, and strongly associated with mitochondrial quality (Meng et al., 2019). SIRT3 mediates FOXO3a activation and the PINK1-Parkin mitophagic induction to prevent cellular death (Jacobs et al., 2008; Li et al., 2018; Zhang et al., 2020). SIRT3 activation alleviates Aβ toxicity and repairs mitochondrial bioenergetics and maintains mitophagic activity and cognitive function in AD (Meng et al., 2019; Li et al., 2020; Yin et al., 2020; Yu et al., 2020; Zhang et al., 2020; Tyagi et al., 2021). SIRT3 expression was lower in the temporal cortices from *APOE* ε4 carriers than non-carriers (Yin et al., 2020). Additionally, SIRT3 expression is reduced and is rescued with ketones treatment only in *APOE* ε4-TR mice (Yin et al., 2019). Collectively, SIRT3, a downstream modulator of ApoE4, may provide a novel direction for AD pathology-related therapy targeting FOXO3a and PINK1-Parkin signaling.

TOMM40, a channel-forming subunit at OMM constituting the TOMM40 complex for protein import into mitochondria (Humphries et al., 2005; Manczak et al., 2011; Liu et al., 2018), is proposed as a novel biomarker of AD (Mise et al., 2017; Ochi et al., 2020). The TOMM-dependent signaling is well-known as a molecular hinge for PINK1/Parkin-mediated mitophagy (Bertolin et al., 2013). The defective TOMM complex is responsible for PINK1 accumulation and activation, as well as Parkin recruitment *via* direct interaction with TOMM40 (Bertolin et al., 2013). Compared to ApoE3 astrocytes, the protein level of TOMM40 is upregulated without growing transcriptional activity, indicating that augmented TOMM40 level in ApoE4 astrocytes may be on account for damaged degradation (Schmukler et al., 2020). This result is consistence with other findings regarding TOMM40 increase in the N_2_a cells by proteomic profiling (Mise et al., 2017) and in both hippocampal homogenates and CA3 pyramidal cells from ApoE4 mice by immunoblot (Orr et al., 2019). Owing to the linkage disequilibrium with *APOE* (Simonovitch et al., 2019), *TOMM40* expression is closely associated with *APOE* expression (Mise et al., 2017), and *APOE*-*TOMM40*-*APOC1* variants are strongly associated with a high instance of AD (Gottschalk et al., 2014; Kulminski et al., 2022). With increasing age, *APOE* ε4 has closely linked to various *TOMM40* polymorphisms carried adverse impacts (Roses et al., 2013; Li et al., 2022), and alter the distribution and function of the TOMM40 haplotypes (Roses et al., 2010). which echoes the mentioned findings—more FL-PINK1 and Parkin, less cleaved PINK1 caused by ApoE4 (Simonovitch et al., 2019). Overall, TOMM40 polymorphisms and expression are associated with *APOE* ε4, and its degradation is blocked under an ApoE4-induced context, which may be helpful to explore the underlying mechanisms of AD.

VDAC1, a porin protein abundantly located at OMM that mediates ATP production and the trafficking of nucleotide and metabolites, functions as a target for Parkin-directed poly-ubiquitin chains, and regulates Parkin recruitment (Sun et al., 2012), thus playing an indispensable role in PINK1/Parkin-mediated mitophagy (Geisler et al., 2010; Yamaguchi et al., 2016) and AD pathogenesis (Manczak and Reddy, 2012b; Shoshan-Barmatz et al., 2018; Chi et al., 2021). VDAC1 preliminarily showed an increase in ApoE4-N_2_a cells performed by proteomic profiling assays (Orr et al., 2019), which is appealed to further experimental verification. Interestingly, a recent study reports the binding of ApoE and VDAC induces the continual opening of the mitochondrial permeability transition pore thus resulting in MMP collapse and electronegative LDL-mediated mitochondrial disorders and impaired degradation (Chen et al., 2020), which may partially account for the VDAC1 elevation abovementioned (Orr et al., 2019). Specifically, it is shown both ApoE3 and ApoE4 are colocalized with VDAC *via* double-labeling analysis, and reconfirmed the interaction without isoform-dependent difference *via* co-IP in H9c2 cells, a type of embryonic rat heart-derived cells (Chen et al., 2020). Although discrepant interaction between ApoE3 and ApoE4 could not be found, the finding enlighten the researchers to broadly examine the ApoE-VDAC interaction in neuronal models, including cell lines of neurons or glia, primary neuronal cells, and brain tissues from *APOE*-TR mice or AD model mice, even the *APOE* ε4 carriers and AD subjects, in order to explore whether the interaction exists in the neuronal system, whether there is a differential interaction between ApoE3 and ApoE4, and whether it is associated with AD pathogenesis. Incidentally, the VDAC1-Aβ and VDAC1-Tau interaction is confirmed by double-labeling analysis and co-IP in the brains of AD subjects and several AD model mice (Manczak and Reddy, 2012b), while it is well known that ApoE4 mediates Aβ aggregation and Tau hyperphosphorylation (Ellis et al., 1996; Brecht et al., 2004). Looking at the above factors, it is supposed that the ApoE-VDAC binding cooperates with the VDAC1-Aβ and VDAC1-Tau interaction conjointly blocks mitochondrial pores as synergistic effects, ultimately contributing to mitochondrial dysfunction, defective mitophagy, and aberrant accumulation of damaged mitochondria, interrupting energy supply and aggravating synaptic dysfunction in AD.

Aβ aggregation causes reductive cytosolic Parkin and PINK1 levels in the cytoplasm (Kerr et al., 2017); Aβ also binding to Parkin and autophagic vacuoles in the distal axons of APP-mutant mouse cells (Cai and Tammineni, 2016; Reddy and Oliver, 2019). Presenilins mutations interrupt PINK1 elevation and PINK/Parkin-dependent mitophagic cycle (Goudarzi et al., 2021). More Parkin recruitment of cytosolic to depolarized mitochondria is shown in human APP-transgenic neurons (Cai and Tammineni, 2016). Dysfunctional Parkin translocation and recruitment result from Tau accumulation and detrimental interactions with Tau (Goudarzi et al., 2021).

Collectively, ApoE4 and PINK1-Parkin comprise a potential mitochondrial signaling cascade response pathway, and these observations indicate ApoE4-mediated inhibition on PINK1-Parkin mitophagy *via* multi-faced downstream regulators (Sohn et al., 2021). The data provide new insights into the role of ApoE4 in mitophagic deficiency to develop a potential therapeutic target for AD.

## Conclusion and Prospects

Deficient mitochondrial quality control is established to interfere with cellular bioenergetic metabolisms and neuronal survival, leading to the occurrence and development of neurodegenerative disorders (Chinnery, 2015). Based on the multi-faceted roles that mitophagy plays in AD pathogenesis (Salminen et al., 2013), we integrate various overwhelming evidence ranging from cellular models to clinical patients that autophagic and mitophagic dysfunction result from ApoE4—multiple interferences of general autophagic processes, fission inhibition, fusion-related protein accumulation, PINK1/Parkin signaling blockage—and highlight additional findings that helps to further elucidate the cross talk of ApoE4 and its effector, as well as the regulations of mitochondrial dynamics events.

Despite current studies about mitophagic control, some important issues are required to be addressed. First, ApoE4 participates in diverse mitophagic signaling pathways at various processes in direct or indirect manners (Horner et al., 2020; Schmukler et al., 2020; Yin et al., 2020; Liang et al., 2021b; Qi et al., 2021; Eran and Ronit, 2022). Taking the cross talk among these signaling pathways into account, it is proposed to further study the interconnected interactions between different pathways, and distinguish which of them plays a predominant role in the pathological consequences of ApoE4. Moreover, certain factors play multiple roles in mitochondria metabolism. SIRT1 (Lee et al., 2008; Jang et al., 2012; Lu et al., 2014; Ou et al., 2014; Qiao et al., 2018; Yao et al., 2021), Ca^2+^ (Cereghetti et al., 2008; Ishihara et al., 2017; Barazzuol et al., 2020), Aβ (Manczak et al., 2011; Cai and Tammineni, 2016; Kerr et al., 2017; Reddy and Oliver, 2019), and Tau (Manczak and Reddy, 2012a; Abtahi et al., 2020; Goudarzi et al., 2021; Kandimalla et al., 2021) function throughout autophagic general processes and mitochondria-specific processes and affect the balance between mitophagy, mitochondrial biogenesis, and mitochondrial dynamics. Second, concerning high AD prevalence in females, it is recommended to highlight the probable role of gender differences or interference in various behavior assessments or molecular indicators in clinical or non-clinical studies in order to find more meticulous findings underlying the complicated effects of *APOE* genotype. Third, the neuro-degeneration processes may share a common mechanism with AD and other neurodegenerative diseases. ApoE has been extensively studied in multiple fields and displays new findings, such as the interaction of ApoE4-*TFEB* promoter (Parcon et al., 2018), ApoE4-*SIRT1* promoter (Theendakara et al., 2016; Lima et al., 2020), and particularly ApoE-VADC (Chen et al., 2020) in myocardial cells, which deserves extending to neuronal researches, particularly mitophagy in AD. There is a preference for detailed, multivariate, and diversified indicators to investigate molecular and functional alterations in new aspects. From a molecular perspective, it may be worthy to differentiate the features of the specific interactions more detailedly, including specificity, affinity, stability, intermolecular force, and other quality at the submolecular level. Fourth, because only few studies have reported OPA1, FIS1 function, and receptor-mediated mitophagy differed from an *APOE-*genotype perspective, hence, completing a related investigation may be helpful to better understand the complicated alterations in AD (Sun et al., 2014; He et al., 2019). Fifth, mitophagic stimulation may be beneficial to tissue homeostasis, as judged by the neuroprotective implication of rapamycin has been comprehensively assessed (Schmukler et al., 2020). Studies may be intriguing to investigate the clinical benefits of more known autophagic or mitophagic inducers or other drugs, such as GRP75 inhibitor (Liang et al., 2021b), *in vivo* and *in vitro* studies and further develop specific therapies.

In general, mitophagy is a multifunctional event preventing AD pathogenesis along with multiple challenges to its beneficial effects. Current findings of the ApoE4 role of mitophagy in AD seem to be limited to some extent, and remain controversial still need further investigation in detail. Discovering the gaps in the underlying mechanisms are greatly enlightened to screen for possible and predominant mechanisms and open a new avenue of research to facilitate earlier diagnosis or potential neuroprotective therapies for AD.

## Author Contributions

GC had the idea for the article and made the final approval of version to be submitted. HC wrote and critically revised the manuscript the work. YiJ and FC supervised the overall writing and edited the manuscript. GH and LZ performed the literature search, sorting, and integration. FS and MZ provided guidance, helpful discussion, and careful revision. YC and YaJ helped the schematic diagrams. All authors contributed to the article and approved the submitted version.

## Conflict of Interest

The authors declare that the research was conducted in the absence of any commercial or financial relationships that could be construed as a potential conflict of interest.

## Publisher's Note

All claims expressed in this article are solely those of the authors and do not necessarily represent those of their affiliated organizations, or those of the publisher, the editors and the reviewers. Any product that may be evaluated in this article, or claim that may be made by its manufacturer, is not guaranteed or endorsed by the publisher.

## References

[B1] (2020). 2020 Alzheimer's disease facts and figures. Alzheimers Dement. 17, 327–406. 10.1002/alz.12068 33756057

[B2] Abtahi S. Masoudi R. Haddadi M. (2020). The distinctive role of Tau and amyloid beta in mitochondrial dysfunction through alteration in Mfn2 and Drp1 Mrna levels: a comparative study in *Drosophila melanogaster*. Gene 754, 144854. 10.1016/j.gene.2020.144854 32525045

[B3] Ashrafi G. Schwarz T. (2015). Pink1- and Park2-mediated local mitophagy in distal neuronal axons. Autophagy 11, 187–189. 10.1080/15548627.2014.996021 25607607 PMC4502792

[B4] Barazzuol L. Giamogante F. Brini M. Calì T. (2020). Pink1/Parkin mediated mitophagy, Ca signalling, and Er-mitochondria contacts in Parkinson's disease. Int. J. Mol. Sci. 21, 1775. 10.3390/ijms21051772 32150829 PMC7084677

[B5] Belinson H. Lev D. Masliah E. Michaelson D. (2008). Activation of the amyloid cascade in apolipoprotein E4 transgenic mice induces lysosomal activation and neurodegeneration resulting in marked cognitive deficits. J. Neurosci. 28, 4690–4701. 10.1523/jneurosci.5633-07.2008 18448646 PMC3844816

[B6] Belloy M. E. Napolioni V. Han S. S. Guenn Y. L. Greicius M. (2020). Association of klotho -vs heterozygosity with risk of Alzheimer disease in individuals who carry Apoe4. JAMA Neurol. 77, 849–862. 10.1001/jamaneurol.2020.0414 32282020 PMC7154955

[B7] Bertolin G. Ferrando-Miguel R. Jacoupy M. Traver S. Grenier K. Greene A.. (2013). The Tomm machinery is a molecular switch in Pink1 and Park2/Parkin-dependent mitochondrial clearance. Autophagy 9, 1801–1817. 10.4161/auto.25884 24149440

[B8] Bhupana J. Huang B. Liou G. Calkins M. Lin-Chao S. (2020). Gas7 knockout affects Pink1 expression and mitochondrial dynamics in mouse cortical neurons. FASEB Bioadv. 2, 166–181. 10.1096/fba.2019-00091 32161906 PMC7059628

[B9] Bohrmann B. Baumann K. Benz J. Gerber F. Huber W. Knoflach F.. (2012). Gantenerumab: a novel human anti-Aβ antibody demonstrates sustained cerebral amyloid-β binding and elicits cell-mediated removal of human amyloid-β. J. Alzheimers Dis. 28, 49–69. 10.3233/jad-2011-110977 21955818

[B10] Bomasang-Layno E. Bronsther R. (2021). Diagnosis and treatment of Alzheimer's disease:: an update. Delaware J. Public Health 7, 74–85. 10.32481/djph.2021.09.009 34604768 PMC8482985

[B11] Brecht W. Harris F. Chang S. Tesseur I. Yu G. Xu Q.. (2004). Neuron-specific apolipoprotein E4 proteolysis is associated with increased tau phosphorylation in brains of transgenic mice. J Neurosci. 24, 2527–2534. 10.1523/jneurosci.4315-03.2004 15014128 PMC6729489

[B12] Butterfield D. Mattson M. (2020). Apolipoprotein E and oxidative stress in brain with relevance to Alzheimer's disease. Neurobiol. Dis. 138, 104795. 10.1016/j.nbd.2020.104795 32036033 PMC7085980

[B13] Cai Q. Jeong Y. (2020). Mitophagy in Alzheimer's disease and other age-related neurodegenerative diseases. Cells 9:150. 10.3390/cells9010150 31936292 PMC7017092

[B14] Cai Q. Tammineni P. (2016). Alterations in mitochondrial quality control in Alzheimer's disease. Front. Cell. Neurosci. 10, 24. 10.3389/fncel.2016.00024 26903809 PMC4746252

[B15] Cereghetti G. Stangherlin A. Martins de Brito O. Chang C. Blackstone C. Bernardi P.. (2008). Dephosphorylation by calcineurin regulates translocation of Drp1 to Mitochondria. Proc. Natl. Acad. Sci. U.S.A. 105, 15803–15808. 10.1073/pnas.0808249105 18838687 PMC2572940

[B16] Chan D. (2012). Fusion and fission: interlinked processes critical for mitochondrial health. Annu. Rev. Genet. 46, 265–287. 10.1146/annurev-genet-110410-132529 22934639

[B17] Chang C. Blackstone C. (2010). Dynamic regulation of mitochondrial fission through modification of the dynamin-related protein Drp1. Ann. N. Y. Acad. Sci. 1201, 34–39. 10.1111/j.1749-6632.2010.05629.x 20649536 PMC5585781

[B18] Chang S. ran Ma T. Miranda R. Balestra M. Mahley R. Huang Y. (2005). Lipid- and receptor-binding regions of apolipoprotein E4 fragments act in concert to cause mitochondrial dysfunction and neurotoxicity. Proc. Natl. Acad. Sci. U.S.A. 102, 18694–18699. 10.1073/pnas.0508254102 16344479 PMC1311737

[B19] Chen H. Chen F. Zhang M. Chen Y. Cui L. Liang C. (2021). A review of apoe genotype-dependent autophagic flux regulation in Alzheimer's disease. J. Alzheimers Dis. 84, 535–555. 10.3233/jad-210602 34569952

[B20] Chen H. Ji Z. Dodson S. Miranda R. Rosenblum C. Reynolds I.. (2011). Apolipoprotein E4 domain interaction mediates detrimental effects on mitochondria and is a potential therapeutic target for Alzheimer disease. J. Biol. Chem. 286, 5215–5221. 10.1074/jbc.M110.151084 21118811 PMC3037634

[B21] Chen M. Chen Z. Wang Y. Tan Z. Zhu C. Li Y.. (2016). Mitophagy receptor Fundc1 regulates mitochondrial dynamics and mitophagy. Autophagy 12, 689–702. 10.1080/15548627.2016.1151580 27050458 PMC4836026

[B22] Chen W. Chen Y. Chan H. Chung C. Peng H. Ho Y.. (2020). Role of apolipoprotein E in electronegative low-density lipoprotein-induced mitochondrial dysfunction in cardiomyocytes. Metab. Clin. Exp. 107, 154227. 10.1016/j.metabol.2020.154227 32275974

[B23] Cheng Z. (2019). The foxo-autophagy axis in health and disease. Trends Endocrinol. Metab. 30, 658–671. 10.1016/j.tem.2019.07.009 31443842

[B24] Chi H. Zhai Q. Zhang M. Su D. Cao W. Li W.. (2021). App/Ps1 gene-environment noise interaction aggravates Ad-like neuropathology in hippocampus via activation of the Vdac1 positive feedback loop. Curr. Alzheimer Res 18, 14–24. 10.2174/1567205018666210324114153 33761858

[B25] Chinnery P. (2015). Mitochondrial disease in adults: what's old and what's new? EMBO Mol. Med. 7, 1503–1512. 10.15252/emmm.201505079 26612854 PMC4693502

[B26] Dhapola R. Sarma P. Medhi B. Prakash A. Reddy D. (2022). Recent advances in molecular pathways and therapeutic implications targeting mitochondrial dysfunction for Alzheimer's disease. Mol. Neurobiol. 59, 535–555. 10.1007/s12035-021-02612-6 34725778

[B27] Du F. Yu Q. Yan S. (2021). Pink1 activation attenuates impaired neuronal-like differentiation and synaptogenesis and mitochondrial dysfunction in Alzheimer's disease trans-mitochondrial cybrid cells. J. Alzheimers Dis. 81, 1749–1761. 10.3233/jad-210095 33998543 PMC9004622

[B28] Egan D. Shackelford D. Mihaylova M. Gelino S. Kohnz R. Mair W.. (2011). Phosphorylation of Ulk1 (Hatg1) by Amp-activated protein kinase connects energy sensing to mitophagy. Science 331, 456–461. 10.1126/science.1196371 21205641 PMC3030664

[B29] Eijkelenboom A. Burgering B. (2013). Foxos: signalling integrators for homeostasis maintenance. Nat. Rev. Mol. Cell Biol. 14, 83–97. 10.1038/nrm3507 23325358

[B30] Eisner V. Picard M. Hajnóczky G. (2018). Mitochondrial dynamics in adaptive and maladaptive cellular stress responses. Nat. Cell Biol. 20, 755–765. 10.1038/s41556-018-0133-0 29950571 PMC6716149

[B31] El Gaamouch F. Jing P. Xia J. Cai D. (2016). Alzheimer's disease risk genes and lipid regulators. J. Alzheimers Dis. 53, 15–29. 10.3233/jad-160169 27128373

[B32] Ellis R. Olichney J. Thal L. Mirra S. Morris J. Beekly D.. (1996). Cerebral amyloid angiopathy in the brains of patients with Alzheimer's Disease: the cerad experience, part XV. Neurology 46, 1592–1596. 10.1212/wnl.46.6.1592 8649554

[B33] Eran S. Ronit P. (2022). Apoe4 expression is associated with impaired autophagy and mitophagy in astrocytes. Neural Regen. Res. 17, 777–778. 10.4103/1673-5374.322452 34472467 PMC8530113

[B34] Fang E. Hou Y. Palikaras K. Adriaanse B. Kerr J. Yang B.. (2019). Mitophagy inhibits amyloid-β and tau pathology and reverses cognitive deficits in models of Alzheimer's disease. Nat. Neurosci. 22, 401–412. 10.1038/s41593-018-0332-9 30742114 PMC6693625

[B35] Fang E. Scheibye-Knudsen M. Brace L. Kassahun H. SenGupta T. Nilsen H.. (2014). Defective mitophagy in Xpa Via Parp-1 hyperactivation and Nad(+)/Sirt1 reduction. Cell 157, 882–896. 10.1016/j.cell.2014.03.026 24813611 PMC4625837

[B36] Fivenson E. Lautrup S. Sun N. Scheibye-Knudsen M. Stevnsner T. Nilsen H.. (2017). Mitophagy in neurodegeneration and aging. Neurochem. Int. 109, 202–209. 10.1016/j.neuint.2017.02.007 28235551 PMC5565781

[B37] Fote G. Geller N. Efstathiou N. Hendricks N. Vavvas D. Reidling J.. (2022). Isoform-dependent lysosomal degradation and internalization of apolipoprotein E requires autophagy proteins. J. Cell Sci. 135:jcs258687. 10.1242/jcs.258687 34982109 PMC8917355

[B38] Fujita Y. Yamashita T. (2018). Sirtuins in neuroendocrine regulation and neurological diseases. Front. Neurosci. 12, 778. 10.3389/fnins.2018.00778 30416425 PMC6213750

[B39] Gao A. Jiang J. Xie F. Chen L. (2020). Bnip3 in mitophagy: novel insights and potential therapeutic target for diseases of secondary mitochondrial dysfunction. Clin. Chim. Acta 506, 72–83. 10.1016/j.cca.2020.02.024 32092316

[B40] Geisler S. Holmström K. Skujat D. Fiesel F. Rothfuss O. Kahle P.. (2010). Pink1/parkin-mediated mitophagy is dependent on Vdac1 and P62/Sqstm1. Nat. Cell Biol. 12, 119–131. 10.1038/ncb2012 20098416

[B41] Gilat-Frenkel M. Boehm-Cagan A. Liraz O. Xian X. Herz J. Michaelson D. (2014). Involvement of the Apoer2 and Lrp1 receptors in mediating the pathological effects of Apoe4 *in vivo*. Curr. Alzheimer Res. 11, 549–557. 10.2174/1567205010666131119232444 24251389 PMC4065855

[B42] Gomes B. Silva J. Romeiro C. Dos Santos S. Rodrigues C. Gonçalves P.. (2018). Neuroprotective mechanisms of resveratrol in Alzheimer's disease: role of Sirt1. Oxidat. Med. Cell. Longevity 2018, 8152373. 10.1155/2018/8152373 30510627 PMC6232815

[B43] Gomes L. Di Benedetto G. Scorrano L. (2011). During autophagy mitochondria elongate, are spared from degradation and sustain cell viability. Nat. Cell Biol. 13, 589–598. 10.1038/ncb2220 21478857 PMC3088644

[B44] Gottschalk W. Lutz M. He Y. Saunders A. Burns D. Roses A.. (2014). The broad impact of Tom40 on neurodegenerative diseases in aging. J. Parkinsons Dis. Alzheimers Dis. 1:12. 10.13188/2376-922x.1000003 25745640 PMC4346331

[B45] Goudarzi S. Hosseini A. Abdollahi M. Haghi-Aminjan H. (2021). Insights into Parkin-mediated mitophagy in Alzheimer's disease: a systematic review. Front. Aging Neurosci. 13, 674071. 10.3389/fnagi.2021.674071 34393755 PMC8358451

[B46] Greer E. Brunet A. (2005). Foxo transcription factors at the interface between longevity and tumor suppression. Oncogene 24, 7410–7425. 10.1038/sj.onc.1209086 16288288

[B47] Guan R. Zou W. Dai X. Yu X. Liu H. Chen Q.. (2018). Mitophagy, a potential therapeutic target for stroke. J. Biomed. Sci. 25, 87. 10.1186/s12929-018-0487-4 30501621 PMC6271612

[B48] Gustafsson Å. Dorn G. (2019). Evolving and expanding the roles of mitophagy as a homeostatic and pathogenic process. Physiol. Rev. 99, 853–892. 10.1152/physrev.00005.2018 30540226 PMC6442924

[B49] Han Y. Wang N. Kang J. Fang Y. (2020). β-asarone improves learning and memory in Aβ-induced Alzheimer's Disease rats by regulating Pink1-Parkin-mediated mitophagy. Metab. Brain Dis. 35, 1109–1117. 10.1007/s11011-020-00587-2 32556928

[B50] He C. Lu S. Wang X. Wang C. Wang L. Liang S.. (2021). Foxo3a protects glioma cells against temozolomide-induced DNA double strand breaks via promotion of Bnip3-mediated mitophagy. Acta Pharmacol. Sin. 42, 1324–1337. 10.1038/s41401-021-00663-y 33879840 PMC8285492

[B51] He L. Zhou Q. Huang Z. Xu J. Zhou H. Lv D.. (2019). Pink1/Parkin-mediated mitophagy promotes Apelin-13-induced vascular smooth muscle cell proliferation by Ampkα and exacerbates atherosclerotic lesions. J. Cell. Physiol. 234, 8668–8682. 10.1002/jcp.27527 30456860

[B52] Hoffman J. Yanckello L. Chlipala G. Hammond T. McCulloch S. Parikh I.. (2019). Dietary inulin alters the gut microbiome, enhances systemic metabolism and reduces neuroinflammation in an Apoe4 mouse model. PLoS ONE 14, e0221828. 10.1371/journal.pone.0221828 31461505 PMC6713395

[B53] Holness M. Sugden M. (2003). Regulation of pyruvate dehydrogenase complex activity by reversible phosphorylation. Biochem. Soc. Trans. 31, 1143–1151. 10.1042/bst0311143 14641014

[B54] Horner S. Berger L. Gibas K. (2020). Nutritional ketosis and photobiomodulation remediate mitochondria warding off Alzheimer's disease in a diabetic, Apoe4+ patient with mild cognitive impairment: a case report. Photodiagn. Photodyn. Therapy 30, 101777. 10.1016/j.pdpdt.2020.101777 32305654

[B55] Huang Y. Liu X. Wyss-Coray T. Brecht W. Sanan D. Mahley R. (2001). Apolipoprotein E fragments present in Alzheimer's disease brains induce neurofibrillary tangle-like intracellular inclusions in neurons. Proc. Natl. Acad. Sci. U.S.A. 98, 8838–8843. 10.1073/pnas.151254698 11447277 PMC37522

[B56] Humphries A. Streimann I. Stojanovski D. Johnston A. Yano M. Hoogenraad N.. (2005). Dissection of the mitochondrial import and assembly pathway for human Tom40. J. Biol. Chem. 280, 11535–11543. 10.1074/jbc.M413816200 15644312

[B57] Ishihara N. Maeda M. Ban T. Mihara K. (2017). Cell-free mitochondrial fusion assay detected by specific protease reaction revealed Ca2+ as regulator of mitofusin-dependent mitochondrial fusion. J. Biochem. 162, 287–294. 10.1093/jb/mvx029 28460043

[B58] Jabeen K. Rehman K. Akash M. (2021). Genetic mutations of Apoeε4 carriers in cardiovascular patients lead to the development of insulin resistance and risk of Alzheimer's disease. J. Biochem. Mol. Toxicol. 2021, e22953. 10.1002/jbt.22953 34757642

[B59] Jacobs K. Pennington J. Bisht K. Aykin-Burns N. Kim H. Mishra M.. (2008). Sirt3 interacts with the Daf-16 homolog Foxo3a in the mitochondria, as well as increases Foxo3a dependent gene expression. Int. J. Biol. Sci. 4, 291–299. 10.7150/ijbs.4.291 18781224 PMC2532794

[B60] Jang S. Kang H. Hwang E. (2012). Nicotinamide-induced mitophagy: event mediated by high Nad+/Nadh ratio and Sirt1 protein activation. J. Biol. Chem. 287, 19304–19314. 10.1074/jbc.M112.363747 22493485 PMC3365962

[B61] Ji Z. Miranda R. Newhouse Y. Weisgraber K. Huang Y. Mahley R. (2002). Apolipoprotein E4 potentiates amyloid beta peptide-induced lysosomal leakage and apoptosis in neuronal cells. J. Biol. Chem. 277, 21821–21828. 10.1074/jbc.M112109200 11912196

[B62] Jin S. Lazarou M. Wang C. Kane L. Narendra D. Youle R. (2010). Mitochondrial membrane potential regulates Pink1 import and proteolytic destabilization by Parl. J. Cell Biol. 191, 933–942. 10.1083/jcb.201008084 21115803 PMC2995166

[B63] Julien C. Tremblay C. Emond V. Lebbadi M. Salem N. Bennett D.. (2009). Sirtuin 1 reduction parallels the accumulation of tau in Alzheimer disease. J. Neuropathol. Exp. Neurol. 68, 48–58. 10.1097/NEN.0b013e3181922348 19104446 PMC2813570

[B64] Kandimalla R. Manczak M. Fry D. Suneetha Y. Sesaki H. Reddy P. (2016). Reduced dynamin-related protein 1 protects against phosphorylated tau-induced mitochondrial dysfunction and synaptic damage in Alzheimer's disease. Hum Mol. Genet. 25, 4881–4897. 10.1093/hmg/ddw312 28173111 PMC6078590

[B65] Kandimalla R. Manczak M. Pradeepkiran J. Morton H. Reddy P. (2021). A partial reduction of Drp1 improves cognitive behavior and enhances mitophagy, autophagy and dendritic spines in a transgenic tau mouse model of Alzheimer disease. Hum Mol. Genet. ddab360. 10.1093/hmg/ddab360 34919689 PMC9169458

[B66] Kauppila T. Kauppila J. Larsson N. (2017). Mammalian mitochondria and aging: an update. Cell Metab. 25, 57–71. 10.1016/j.cmet.2016.09.017 28094012

[B67] Kaur D. Sharma V. Deshmukh R. (2019). Activation of microglia and astrocytes: a roadway to neuroinflammation and Alzheimer's disease. Inflammopharmacology 27, 663–677. 10.1007/s10787-019-00580-x 30874945

[B68] Kerr J. Adriaanse B. Greig N. Mattson M. Cader M. Bohr V.. (2017). Mitophagy and Alzheimer's disease: cellular and molecular mechanisms. Trends Neurosci. 40, 151–166. 10.1016/j.tins.2017.01.002 28190529 PMC5341618

[B69] Kothari S. Bala N. Patel A. Donovan A. Narayanaswami V. (2021). The Ldl receptor binding domain of apolipoprotein E directs the relative orientation of its C-terminal segment in reconstituted nascent Hdl. Biochim Biophys Acta Biomemb. 1863, 183618. 10.1016/j.bbamem.2021.183618 33831404 PMC8211829

[B70] Kulminski A. Philipp I. Shu L. Culminskaya I. (2022). Definitive roles of Tomm40-Apoe-Apoc1 variants in the Alzheimer's risk. Neurobiol. Aging 110, 122–131. 10.1016/j.neurobiolaging.2021.09.009 34625307 PMC8758518

[B71] Kuruva C. Manczak M. Yin X. Ogunmokun G. Reddy A. Reddy P. (2017). Aqua-soluble Ddq reduces the levels of Drp1 and Aβ and inhibits abnormal interactions between Aβ and Drp1 and protects Alzheimer's disease neurons from Aβ- and Drp1-induced mitochondrial and synaptic toxicities. Hum Mol. Genet. 26, 3375–3395. 10.1093/hmg/ddx226 28854701 PMC5886305

[B72] Larramona-Arcas R. González-Arias C. Perea G. Gutiérrez A. Vitorica J. García-Barrera T.. (2020). Sex-dependent calcium hyperactivity due to lysosomal-related dysfunction in astrocytes from Apoe4 versus Apoe3 gene targeted replacement mice. Mol. Neurodegen. 15, 35. 10.1186/s13024-020-00382-8 32517777 PMC7285605

[B73] Lattanzio F. Carboni L. Carretta D. Rimondini R. Candeletti S. Romualdi P. (2014). Human apolipoprotein E4 modulates the expression of Pin1, Sirtuin 1, and Presenilin 1 in brain regions of targeted replacement apoe mice. Neuroscience 256, 360–369. 10.1016/j.neuroscience.2013.10.017 24161275

[B74] Lee I. Cao L. Mostoslavsky R. Lombard D. Liu J. Bruns N.. (2008). A role for the Nad-dependent deacetylase Sirt1 in the regulation of autophagy. Proc. Natl. Acad. Sci. U.S.A. 105, 3374–3379. 10.1073/pnas.0712145105 18296641 PMC2265142

[B75] Leng F. Edison P. (2021). Neuroinflammation and microglial activation in Alzheimer disease: where do we go from here? Nat. Rev. Neurol. 17, 157–172. 10.1038/s41582-020-00435-y 33318676

[B76] Levy E. Carman M. Fernandez-Madrid I. Power M. Lieberburg I. van Duinen S.. (1990). Mutation of the Alzheimer's disease amyloid gene in hereditary cerebral hemorrhage, Dutch type. Science 248, 1124–1126. 10.1126/science.2111584 2111584

[B77] Li T. Pappas C. Le S. Wang Q. Klinedinst B. Larsen B.. (2022). Apoe, Tomm40, and sex interactions on neural network connectivity. Neurobiol. Aging 109, 158–165. 10.1016/j.neurobiolaging.2021.09.020 34740077 PMC8694046

[B78] Li W. Su D. Zhai Q. Chi H. She X. Gao X.. (2019). Proteomes analysis reveals the involvement of autophagy in Ad-like neuropathology induced by noise exposure and Apoe4. Environ. Res. 176, 108537. 10.1016/j.envres.2019.108537 31228807

[B79] Li X. Hu Y. Wang Z. Luo Y. Zhang Y. Liu X.. (2016). Human wild-type full-length tau accumulation disrupts mitochondrial dynamics and the functions via increasing mitofusins. Sci. Rep. 6, 24756. 10.1038/srep24756 27099072 PMC4838862

[B80] Li Y. Lu J. Cao X. Zhao H. Gao L. Xia P.. (2020). βa newly synthesized rhamnoside derivative alleviates Alzheimer's amyloid–induced oxidative stress, mitochondrial dysfunction, and cell senescence through upregulating Sirt3. Oxidat. Med. Cell. Longevity 2020, 7698560. 10.1155/2020/7698560 32104538 PMC7040408

[B81] Li Y. Ma Y. Song L. Yu L. Zhang L. Zhang Y.. (2018). Sirt3 deficiency exacerbates P53/Parkin-mediated mitophagy inhibition and promotes mitochondrial dysfunction: implication for aged hearts. Int. J. Mol. Med. 41, 3517–3526. 10.3892/ijmm.2018.3555 29532856

[B82] Li Y. Zheng W. Lu Y. Zheng Y. Pan L. Wu X.. (2021). Bnip3l/Nix-mediated mitophagy: molecular mechanisms and implications for human disease. Cell Death Dis. 13, 14. 10.1038/s41419-021-04469-y 34930907 PMC8688453

[B83] Liang J. Wang C. Zhang H. Huang J. Xie J. Chen N. (2021a). Exercise-induced benefits for Alzheimer's disease by stimulating mitophagy and improving mitochondrial function. Front. Aging Neurosci. 13, 755665. 10.3389/fnagi.2021.755665 34658846 PMC8519401

[B84] Liang T. Hang W. Chen J. Wu Y. Wen B. Xu K.. (2021b). Apoe4 (Δ272-299) induces mitochondrial-associated membrane formation and mitochondrial impairment by enhancing Grp75-modulated mitochondrial calcium overload in neuron. Cell Biosci. 11, 50. 10.1186/s13578-021-00563-y 33676568 PMC7937300

[B85] Lima D. Hacke A. Inaba J. Pessôa C. Kerman K. (2020). Electrochemical detection of specific interactions between apolipoprotein E isoforms and DNA sequences related to Alzheimer's disease. Bioelectrochemistry 133, 107447. 10.1016/j.bioelechem.2019.107447 32006858

[B86] Liu C. Liu C. Kanekiyo T. Xu H. Bu G. (2013). Apolipoprotein E and Alzheimer disease: risk, mechanisms and therapy. Nat. Rev. Neurol. 9, 106–118. 10.1038/nrneurol.2012.263 23296339 PMC3726719

[B87] Liu W. Duan X. Fang X. Shang W. Tong C. (2018). Mitochondrial protein import regulates cytosolic protein homeostasis and neuronal integrity. Autophagy 14, 1293–1309. 10.1080/15548627.2018.1474991 29909722 PMC6208451

[B88] Lu P. Kamboj A. Gibson S. Anderson C. (2014). Poly(Adp-Ribose) polymerase-1 causes mitochondrial damage and neuron death mediated by Bnip3. J. Neurosci. 34, 15975–15987. 10.1523/jneurosci.2499-14.2014 25429139 PMC6608475

[B89] Luan Y. Ren K. Luan Y. Chen X. Yang Y. (2021). Mitochondrial dynamics: pathogenesis and therapeutic targets of vascular diseases. Front. Cardiovasc. Med. 8, 770574. 10.3389/fcvm.2021.770574 34938787 PMC8685340

[B90] Mahaman Y. Embaye K. Huang F. Li L. Zhu F. Wang J.. (2022). Biomarkers used in Alzheimer's disease diagnosis, treatment, and prevention. Ageing Res. Rev. 74, 101544. 10.1016/j.arr.2021.101544 34933129

[B91] Manczak M. Calkins M. Reddy P. (2011). Impaired mitochondrial dynamics and abnormal interaction of amyloid beta with mitochondrial protein Drp1 in neurons from patients with Alzheimer's disease: implications for neuronal damage. Hum. Mol. Genet. 20, 2495–2509. 10.1093/hmg/ddr139 21459773 PMC3109997

[B92] Manczak M. Kandimalla R. Fry D. Sesaki H. Reddy P. (2016). Protective effects of reduced dynamin-related protein 1 against amyloid beta-induced mitochondrial dysfunction and synaptic damage in Alzheimer's disease. Hum. Mol. Genet. 25, 5148–5166. 10.1093/hmg/ddw330 27677309 PMC6078633

[B93] Manczak M. Reddy P. (2012a). Abnormal interaction between the mitochondrial fission protein Drp1 and hyperphosphorylated Tau in Alzheimer's disease neurons: implications for mitochondrial dysfunction and neuronal damage. Hum. Mol. Genet. 21, 2538–2547. 10.1093/hmg/dds072 22367970 PMC3349426

[B94] Manczak M. Reddy P. (2012b). Abnormal interaction of Vdac1 with amyloid beta and phosphorylated tau causes mitochondrial dysfunction in Alzheimer's disease. Hum. Mol Genet. 21, 5131–5146. 10.1093/hmg/dds360 22926141 PMC3490521

[B95] Martín-Maestro P. Gargini R. Perry G. Avila J. García-Escudero V. (2016). Park2 enhancement is able to compensate mitophagy alterations found in sporadic Alzheimer's disease. Hum. Mol. Genet. 25, 792–806. 10.1093/hmg/ddv616 26721933 PMC4743695

[B96] Matsuda N. Sato S. Shiba K. Okatsu K. Saisho K. Gautier C.. (2010). Pink1 stabilized by mitochondrial depolarization recruits parkin to damaged mitochondria and activates latent parkin for mitophagy. J. Cell Biol. 189, 211–221. 10.1083/jcb.200910140 20404107 PMC2856912

[B97] Meng H. Yan W. Lei Y. Wan Z. Hou Y. Sun L.. (2019). Sirt3 regulation of mitochondrial quality control in neurodegenerative diseases. Front. Aging Neurosci. 11, 313. 10.3389/fnagi.2019.00313 31780922 PMC6861177

[B98] Mise A. Yoshino Y. Yamazaki K. Ozaki Y. Sao T. Yoshida T.. (2017). Tomm40 and Apoe gene expression and cognitive decline in Japanese Alzheimer's disease subjects. J. Alzheimers Dis. 60, 1107–1117. 10.3233/jad-170361 28984592

[B99] Misrani A. Tabassum S. Huo Q. Tabassum S. Jiang J. Ahmed A.. (2021). Mitochondrial deficits with neural and social damage in early-stage Alzheimer's disease model mice. Front. Aging Neurosci. 13, 748388. 10.3389/fnagi.2021.748388 34955809 PMC8704997

[B100] Mkrtchyan G. Abdelmohsen K. Andreux P. Bagdonaite I. Barzilai N. Brunak S.. (2020). Ardd 2020: from aging mechanisms to interventions. Aging 12, 24484–24503. 10.18632/aging.202454 33378272 PMC7803558

[B101] Monzio Compagnoni G. Di Fonzo A. Corti S. Comi G. Bresolin N. Masliah E. (2020). The role of mitochondria in neurodegenerative diseases: the lesson from Alzheimer's disease and Parkinson's disease. Mol. Neurobiol. 57, 2959–2980. 10.1007/s12035-020-01926-1 32445085 PMC9047992

[B102] Moreira P. Carvalho C. Zhu X. Smith M. Perry G. (2010). Mitochondrial dysfunction is a trigger of Alzheimer's disease pathophysiology. Biochim. Biophys. Acta 1802, 2–10. 10.1016/j.bbadis.2009.10.006 19853658

[B103] Morton H. Kshirsagar S. Orlov E. Bunquin L. Sawant N. Boleng L.. (2021). Defective mitophagy and synaptic degeneration in Alzheimer's disease: focus on aging, mitochondria and synapse. Free Radical Biol. Med. 172, 652–667. 10.1016/j.freeradbiomed.2021.07.013 34246776

[B104] Motta M. Divecha N. Lemieux M. Kamel C. Chen D. Gu W.. (2004). Mammalian Sirt1 represses forkhead transcription factors. Cell 116, 551–563. 10.1016/s0092-8674(04)00126-6 14980222

[B105] Murtaza G. Khan A. Rashid R. Muneer S. Hasan S. Chen J. (2017). Foxo transcriptional factors and long-term living. Oxidat. Med. Cell. Longevity 2017, 3494289. 10.1155/2017/3494289 28894507 PMC5574317

[B106] Narendra D. Walker J. Youle R. (2012). Mitochondrial quality control mediated by Pink1 and Parkin: links to Parkinsonism. Cold Spring Harbor Perspect. Biol. 4:a011338. 10.1101/cshperspect.a011338 23125018 PMC3536340

[B107] Nguyen T. Padman B. Lazarou M. (2016). Deciphering the molecular signals of Pink1/Parkin mitophagy. Trends Cell Biol. 26, 733–744. 10.1016/j.tcb.2016.05.008 27291334

[B108] Norwitz N. Saif N. Ariza I. Isaacson R. (2021). Apoe4precision nutrition for Alzheimer's prevention in carriers. Nutrients 13:1362. 10.3390/nu13041362 33921683 PMC8073598

[B109] Nuriel T. Peng K. Ashok A. Dillman A. Figueroa H. Apuzzo J.. (2017). Apoe4the endosomal-lysosomal pathway is dysregulated by expression. Front. Neurosci. 11, 702. 10.3389/fnins.2017.00702 29311783 PMC5733017

[B110] Ochi S. Iga J. Funahashi Y. Yoshino Y. Yamazaki K. Kumon H.. (2020). Identifying blood transcriptome biomarkers of Alzheimer's disease using transgenic mice. Mol. Neurobiol. 57, 4941–4951. 10.1007/s12035-020-02058-2 32816243 PMC7541363

[B111] Ong Q. Chan E. Lim M. Wong B. (2014). Expression of human apolipoprotein E4 reduces insulin-receptor substrate 1 expression and Akt phosphorylation in the ageing liver. FEBS Open 4, 260–265. 10.1016/j.fob.2014.02.011 24649407 PMC3958919

[B112] Orr A. Kim C. Jimenez-Morales D. Newton B. Johnson J. Krogan N.. (2019). Neuronal apolipoprotein E4 expression results in proteome-wide alterations and compromises bioenergetic capacity by disrupting mitochondrial function. J. Alzheimers Dis. 68, 991–1011. 10.3233/jad-181184 30883359 PMC6481541

[B113] Ou X. Lee M. Huang X. Messina-Graham S. Broxmeyer H. (2014). Sirt1 positively regulates autophagy and mitochondria function in embryonic stem cells under oxidative stress. Stem Cells 32, 1183–1194. 10.1002/stem.1641 24449278 PMC3991763

[B114] Palikaras K. Daskalaki I. Markaki M. Tavernarakis N. (2017). Mitophagy and age-related pathologies: development of new therapeutics by targeting mitochondrial turnover. Pharmacol. Therapeut. 178, 157–174. 10.1016/j.pharmthera.2017.04.005 28461251

[B115] Pan X. Misrani A. Tabassum S. Yang L. (2021). Mitophagy pathways and Alzheimer's disease: from pathogenesis to treatment. Mitochondrion 59, 37–47. 10.1016/j.mito.2021.04.007 33872797

[B116] Parcon P. Balasubramaniam M. Ayyadevara S. Jones R. Liu L. Shmookler Reis R.. (2018). Apolipoprotein E4 inhibits autophagy gene products through direct, specific binding to clear motifs. Alzheimers Dement. 14, 230–242. 10.1016/j.jalz.2017.07.754 28945989 PMC6613789

[B117] Parker W. Parks J. Filley C. Kleinschmidt-DeMasters B. (1994). Electron transport chain defects in Alzheimer's disease brain. Neurology 44, 1090–1096. 10.1212/wnl.44.6.1090 8208407

[B118] Parzych K. Klionsky D. (2014). An overview of autophagy: morphology, mechanism, and regulation. Antioxid. Redox Signal. 20, 460–473. 10.1089/ars.2013.5371 23725295 PMC3894687

[B119] Persson T. Lattanzio F. Calvo-Garrido J. Rimondini R. Rubio-Rodrigo M. Sundström E.. (2017). Apolipoprotein E4 elicits lysosomal cathepsin D release, decreased thioredoxin-1 levels, and apoptosis. J. Alzheimers Dis. 56, 601–617. 10.3233/jad-150738 28035917 PMC5271484

[B120] Pleen J. Townley R. (2022). Alzheimer's disease clinical trial update 2019-2021. J. Neurol. 269, 1038–1051. 10.1007/s00415-021-10790-5 34609602

[B121] Pradhan R. Yadav S. Prem N. Bhagel V. Pathak M. Shekhar S.. (2020). Serum Foxo3a: a ray of hope for early diagnosis of Alzheimer's disease. Mech. Ageing Dev. 190, 111290. 10.1016/j.mad.2020.111290 32603667

[B122] Qi G. Mi Y. Shi X. Gu H. Brinton R. Yin F. (2021). Apoe4 impairs neuron-astrocyte coupling of fatty acid metabolism. Cell Rep. 34, 108572. 10.1016/j.celrep.2020.108572 33406436 PMC7837265

[B123] Qiao H. Ren H. Du H. Zhang M. Xiong X. Lv R. (2018). Liraglutide repairs the infarcted heart: the role of the Sirt1/Parkin/Mitophagy pathway. Mol. Med. Rep. 17, 3722–3734. 10.3892/mmr.2018.8371 29328405 PMC5802177

[B124] Rambold A. Kostelecky B. Elia N. Lippincott-Schwartz J. (2011). Tubular network formation protects mitochondria from autophagosomal degradation during nutrient starvation. Proc. Natl. Acad. Sci. U.S.A. 108, 10190–10195. 10.1073/pnas.1107402108 21646527 PMC3121813

[B125] Reddy P. Oliver D. (2019). Amyloid beta and phosphorylated tau-induced defective autophagy and mitophagy in Alzheimer's disease. Cells 8:488. 10.3390/cells8050488 31121890 PMC6562604

[B126] Reddy P. Reddy T. Manczak M. Calkins M. Shirendeb U. Mao P. (2011). Dynamin-related protein 1 and mitochondrial fragmentation in neurodegenerative diseases. Brain Res. Rev. 67, 103–118. 10.1016/j.brainresrev.2010.11.004 21145355 PMC3061980

[B127] Reddy P. Yin X. Manczak M. Kumar S. Pradeepkiran J. Vijayan M.. (2018). Mutant app and amyloid beta-induced defective autophagy, mitophagy, mitochondrial structural and functional changes and synaptic damage in hippocampal neurons from Alzheimer's disease. Hum. Mol. Genet. 27, 2502–2516. 10.1093/hmg/ddy154 29701781 PMC6031001

[B128] Rhea E. Banks W. (2021). Interactions of lipids, lipoproteins, and apolipoproteins with the blood-brain barrier. Pharmaceut. Res. 38, 1469–1475. 10.1007/s11095-021-03098-6 34518942 PMC8901411

[B129] Rhodes E. Insel P. Butters M. Morin R. Bickford D. Tosun D.. (2021). The impact of amyloid burden and apoe on rates of cognitive impairment in late life depression. J Alzheimers Dis. 80, 991–1002. 10.3233/jad-201089 33682706 PMC8935860

[B130] Roses A. Lutz M. Amrine-Madsen H. Saunders A. Crenshaw D. Sundseth S.. (2010). A Tomm40 variable-length polymorphism predicts the age of late-onset Alzheimer's disease. Pharmacogenom. J. 10, 375–384. 10.1038/tpj.2009.69 20029386 PMC2946560

[B131] Roses A. Lutz M. Crenshaw D. Grossman I. Saunders A. Gottschalk W. (2013). Tomm40 and Apoe: requirements for replication studies of association with age of disease onset and enrichment of a clinical trial. Alzheimers Dement. 9, 132–136. 10.1016/j.jalz.2012.10.009 23333464

[B132] Salloway S. Sperling R. Fox N. Blennow K. Klunk W. Raskind M.. (2014). Two Phase 3 trials of bapineuzumab in mild-to-moderate Alzheimer's disease. N. Engl. J. Med. 370, 322–333. 10.1056/NEJMoa1304839 24450891 PMC4159618

[B133] Salminen A. Kaarniranta K. (2009). Sirt1: regulation of longevity via autophagy. Cell. Signal. 21, 1356–1360. 10.1016/j.cellsig.2009.02.014 19249351

[B134] Salminen A. Kaarniranta K. Kauppinen A. Ojala J. Haapasalo A. Soininen H.. (2013). Impaired autophagy and app processing in Alzheimer's disease: the potential role of Beclin 1 interactome. Prog. Neurobiol. 106–107, 33–54. 10.1016/j.pneurobio.2013.06.002 23827971

[B135] Sandoval H. Thiagarajan P. Dasgupta S. Schumacher A. Prchal J. Chen M.. (2008). Essential role for nix in autophagic maturation of erythroid cells. Nature 454, 232–235. 10.1038/nature07006 18454133 PMC2570948

[B136] Schmidt M. Gan Z. Komander D. Dewson G. (2021). Ubiquitin signalling in neurodegeneration: mechanisms and therapeutic opportunities. Cell Death Diff. 28, 570–590. 10.1038/s41418-020-00706-7 33414510 PMC7862249

[B137] Schmukler E. Solomon S. Simonovitch S. Goldshmit Y. Wolfson E. Michaelson D.. (2020). Altered mitochondrial dynamics and function in Apoe4-expressing astrocytes. Cell Death Dis. 11, 578. 10.1038/s41419-020-02776-4 32709881 PMC7382473

[B138] Scrivo A. Bourdenx M. Pampliega O. Cuervo A. (2018). Selective autophagy as a potential therapeutic target for neurodegenerative disorders. Lancet Neurol. 17, 802–815. 10.1016/s1474-4422(18)30238-2 30129476 PMC6359907

[B139] Serrano-Pozo A. Das S. Hyman B. (2021). Apoe and Alzheimer's disease: advances in genetics, pathophysiology, and therapeutic approaches. Lancet Neurol. 20, 68–80. 10.1016/s1474-4422(20)30412-9 33340485 PMC8096522

[B140] Shoshan-Barmatz V. Nahon-Crystal E. Shteinfer-Kuzmine A. Gupta R. (2018). Vdac1, mitochondrial dysfunction, and Alzheimer's disease. Pharmacol. Res. 131, 87–101. 10.1016/j.phrs.2018.03.010 29551631

[B141] Simonovitch S. Schmukler E. Bespalko A. Iram T. Frenkel D. Holtzman D.. (2016). Impaired autophagy in Apoe4 astrocytes. J. Alzheimers Dis. 51, 915–927. 10.3233/jad-151101 26923027

[B142] Simonovitch S. Schmukler E. Masliah E. Pinkas-Kramarski R. Michaelson D. (2019). The effects of Apoe4 on mitochondrial dynamics and proteins *in vivo*. J. Alzheimers Dis. 70, 861–875. 10.3233/jad-190074 31306119 PMC7478177

[B143] Sohn H. Kim S. Park J. Park S. Koh Y. Kim J.. (2021). Apoe4 attenuates autophagy via Foxo3a repression in the brain. Sci. Rep. 11, 17604. 10.1038/s41598-021-97117-6 34475505 PMC8413297

[B144] Song M. Zhao X. Song F. (2021). Aging-dependent mitophagy dysfunction in Alzheimer's disease. Mol. Neurobiol. 58, 2362–2378. 10.1007/s12035-020-02248-y 33417222

[B145] Sukhorukov V. Voronkov D. Baranich T. Mudzhiri N. Magnaeva A. Illarioshkin S. (2021). Impaired mitophagy in neurons and glial cells during aging and age-related disorders. Int. J. Mol. Sci. 22:10251. 10.3390/ijms221910251 34638589 PMC8508639

[B146] Sun R. Wang X. Liu Y. Xia M. (2014). Dietary supplementation with fish oil alters the expression levels of proteins governing mitochondrial dynamics and prevents high-fat diet-induced endothelial dysfunction. Brit. J. Nutr. 112, 145–153. 10.1017/s0007114514000701 24775220

[B147] Sun Y. Vashisht A. Tchieu J. Wohlschlegel J. Dreier L. (2012). Voltage-dependent anion channels (Vdacs) recruit parkin to defective mitochondria to promote mitochondrial autophagy. J. Biol. Chem. 287, 40652–40660. 10.1074/jbc.M112.419721 23060438 PMC3504778

[B148] Taguchi N. Ishihara N. Jofuku A. Oka T. Mihara K. (2007). Mitotic phosphorylation of dynamin-related Gtpase Drp1 participates in mitochondrial fission. J. Biol. Chem. 282, 11521–11529. 10.1074/jbc.M607279200 17301055

[B149] Tang M. Ryman D. C. Mcdade E. Jasielec M. S. Buckles V. D. Cairns N. J.. (2016). Neurological manifestations of autosomal dominant Alzheimer's disease from the dian cohort and a meta-analysis. Lancet Neurol. 15, 1317–1325. 10.1016/S1474-4422(16)30229-0 27777020 PMC5116769

[B150] Theendakara V. Patent A. Peters Libeu C. Philpot B. Flores S. Descamps O.. (2013). Neuroprotective sirtuin ratio reversed by Apoe4. Proc. Natl. Acad. Sci. U.S.A. 110, 18303–18308. 10.1073/pnas.1314145110 24145446 PMC3831497

[B151] Theendakara V. Peters-Libeu C. Spilman P. Poksay K. Bredesen D. Rao R. (2016). Direct transcriptional effects of apolipoprotein E. J. Neurosci. 36, 685–700. 10.1523/jneurosci.3562-15.2016 26791201 PMC4719010

[B152] Tilokani L. Nagashima S. Paupe V. Prudent J. (2018). Mitochondrial dynamics: overview of molecular mechanisms. Essays Biochem. 62, 341–360. 10.1042/ebc20170104 30030364 PMC6056715

[B153] Tran M. Reddy P. (2020). Defective autophagy and mitophagy in aging and Alzheimer's disease. Front Neurosci. 14, 612757. 10.3389/fnins.2020.612757 33488352 PMC7820371

[B154] Tyagi A. Mirita C. Shah I. Reddy P. Pugazhenthi S. (2021). Effects of lipotoxicity in brain microvascular endothelial cells during Sirt3 deficiency-potential role in comorbid Alzheimer's disease. Front. Aging Neurosci. 13, 716616. 10.3389/fnagi.2021.716616 34393764 PMC8355826

[B155] Wallon D. Rousseau S. Rovelet-Lecrux A. Quillard-Muraine M. Guyant-Maréchal L. Martinaud O.. (2012). The French series of autosomal dominant early onset Alzheimer's disease cases: mutation spectrum and cerebrospinal fluid biomarkers. J. Alzheimers Dis. 30, 847–856. 10.3233/jad-2012-120172 22475797

[B156] Wang S. Ichinomiya T. Terada Y. Wang D. Patel H. Head B. (2021a). Synapsin-promoted caveolin-1 overexpression maintains mitochondrial morphology and function in Psapp Alzheimer's disease mice. Cells 10:2487. 10.3390/cells10092487 34572135 PMC8467690

[B157] Wang W. Han R. He H. Wang Z. Luan X. Li J.. (2021b). Delineating the role of mitophagy inducers for Alzheimer disease patients. Aging Dis. 12, 852–867. 10.14336/ad.2020.0913 34094647 PMC8139196

[B158] Wang X. Su B. Lee H. Li X. Perry G. Smith M.. (2009). Impaired balance of mitochondrial fission and fusion in Alzheimer's disease. J. Neurosci. 29, 9090–9103. 10.1523/jneurosci.1357-09.2009 19605646 PMC2735241

[B159] Ward A. Crean S. Mercaldi C. Collins J. Boyd D. Cook M.. (2012). Prevalence of apolipoprotein E4 genotype and homozygotes (Apoe E4/4) among patients diagnosed with Alzheimer's disease: a systematic review and meta-analysis. Neuroepidemiology 38, 1–17. 10.1159/000334607 22179327

[B160] Wissler Gerdes E. Zhu Y. Weigand B. Tripathi U. Burns T. Tchkonia T.. (2020). Cellular senescence in aging and age-related diseases: implications for neurodegenerative diseases. Int. Rev. Neurobiol. 155, 203–234. 10.1016/bs.irn.2020.03.019 32854855 PMC7656525

[B161] Wolf C. López Del Amo V. Arndt S. Bueno D. Tenzer S. Hanschmann E.. (2020). Redox modifications of proteins of the mitochondrial fusion and fission machinery. Cells 9:815. 10.3390/cells9040815 32230997 PMC7226787

[B162] Xu L. Shen Y. Wang X. Wei L. Wang P. Yang H.. (2017). Mitochondrial dynamics changes with age in an Appsw/Ps1de9 mouse model of Alzheimer's disease. Neuroreport 28, 222–228. 10.1097/wnr.0000000000000739 28118288 PMC5321113

[B163] Yamaguchi O. Murakawa T. Nishida K. Otsu K. (2016). Receptor-mediated mitophagy. J. Mol. Cell. Cardiol. 95, 50–56. 10.1016/j.yjmcc.2016.03.010 27021519

[B164] Yao J. Wang J. Xu Y. Guo Q. Sun Y. Liu J.. (2021). Cdk9 inhibition blocks the initiation of Pink1-Prkn-mediated mitophagy by regulating the Sirt1-Foxo3-Bnip3 axis and enhances the therapeutic effects involving mitochondrial dysfunction in hepatocellular carcinoma. Autophagy 17, 1–19. 10.1080/15548627.2021.2007027 34890308 PMC9450969

[B165] Yin J. Nielsen M. Li S. Shi J. (2019). Ketones improves apolipoprotein E4-related memory deficiency via sirtuin 3. Aging 11, 4579–4586. 10.18632/aging.102070 31280254 PMC6660057

[B166] Yin J. Reiman E. Beach T. Serrano G. Sabbagh M. Nielsen M.. (2020). Effect of apoe isoforms on mitochondria in Alzheimer disease. Neurology 94, e2404. 10.1212/wnl.0000000000009582 32457210 PMC7455369

[B167] Yu W. Lyu J. Jia L. Sheng M. Yu H. Du H. (2020). Dexmedetomidine ameliorates hippocampus injury and cognitive dysfunction induced by hepatic ischemia/reperfusion by activating Sirt3-mediated mitophagy and inhibiting activation of the Nlrp3 inflammasome in young rats. Oxidat. Med. Cell. Longevity 2020, 7385458. 10.1155/2020/7385458 34493950 PMC8418694

[B168] Zachari M. Ktistakis N. (2020). Mammalian mitophagosome formation: a focus on the early signals and steps. Front. Cell Dev. Biol. 8, 171. 10.3389/fcell.2020.00171 32258042 PMC7093328

[B169] Zhang J. Lin L. Dai X. Xiao N. Ye Q. Chen X. (2021). Apoe4 increases susceptibility to stress-induced age-dependent depression-like behavior and cognitive impairment. J. Psychiatr. Res. 143, 292–301. 10.1016/j.jpsychires.2021.09.029 34530340

[B170] Zhang J. Xiang H. Liu J. Chen Y. He R. Liu B. (2020). Mitochondrial Sirtuin 3: new emerging biological function and therapeutic target. Theranostics 10, 8315–8342. 10.7150/thno.45922 32724473 PMC7381741

[B171] Zhang Q. Xie C. (2021). Apolipoprotein E drives early blood-brain barrier damage in Alzheimer's disease. Neurosci. Bull. 37, 281–283. 10.1007/s12264-020-00596-2 33068281 PMC7870727

[B172] Zhang T. Xue L. Li L. Tang C. Wan Z. Wang R.. (2016). Bnip3 protein suppresses Pink1 kinase proteolytic cleavage to promote mitophagy. J. Biol. Chem. 291, 21616–21629. 10.1074/jbc.M116.733410 27528605 PMC5076832

[B173] Zhao J. Fu Y. Yamazaki Y. Ren Y. Davis M. Liu C.. (2020). Apoe4 exacerbates synapse loss and neurodegeneration in Alzheimer's disease patient Ipsc-derived cerebral organoids. Nat. Commun. 11, 5540. 10.1038/s41467-020-19264-0 33139712 PMC7608683

[B174] Zhu L. Zhong M. Elder G. Sano M. Holtzman D. Gandy S.. (2015). Phospholipid dysregulation contributes to Apoe4-associated cognitive deficits in Alzheimer's disease pathogenesis. Proc. Natl. Acad. Sci. U.S.A. 112, 11965–11970. 10.1073/pnas.1510011112 26372964 PMC4586834

[B175] Zhu X. Perry G. Smith M. Wang X. (2013). Abnormal mitochondrial dynamics in the pathogenesis of Alzheimer's disease. J. Alzheimers Dis. 33, S253–S262. 10.3233/jad-2012-129005 22531428 PMC4097015

